# The Evolution of White Etching Cracks (WECs) in Rolling Contact Fatigue-Tested 100Cr6 Steel

**DOI:** 10.1007/s11249-017-0946-1

**Published:** 2017-11-27

**Authors:** A. D. Richardson, M.-H. Evans, L. Wang, R. J. K. Wood, M. Ingram, B. Meuth

**Affiliations:** 1 nCATS, Faculty of Engineering and the Environment, University of Southampton, Southampton, UK; 2 Afton Chemical Ltd, Bracknell, UK

**Keywords:** White etching cracks, Wind turbine gearbox bearings, White etching area, Rolling contact fatigue, Rolling element bearings, Bearing failure

## Abstract

The formation of white etching cracks (WECs) in steel rolling element bearings can lead to the premature rolling contact fatigue (RCF) failure mode called white structure flaking. Driving mechanisms are still debated but are proposed to be combinations of mechanical, tribochemical and electrical effects. A number of studies have been conducted to record and map WECs in RCF-tested samples and bearings failed from the field. For the first time, this study uses serial sectioning metallography techniques on non-hydrogen charged test samples over a range of test durations to capture the evolution of WEC formation from their initiation to final flaking. Clear evidence for subsurface initiation at non-metallic inclusions was observed at the early stages of WEC formation, and with increasing test duration the propagation of these cracks from the subsurface region to the contact surface eventually causing flaking. In addition, an increase in the amount of associated microstructural changes adjacent to the cracks is observed, this being indicative of the crack being a prerequisite of the microstructural alteration.

**Electronic supplementary material:**

The online version of this article (10.1007/s11249-017-0946-1) contains supplementary material, which is available to authorized users.

## Introduction

Rolling element bearings used in wind turbine gearboxes suffer from a premature failure mode called white structure flaking (WSF). This typically occurs in 1–20% of the bearing’s L_10_ life, where the wind turbine lifetime is reduced from the predicted 20 years to < 2 years [1, 2]. WSF is due to the formation of white etching cracks (WECs) typically ~ 1 mm below the contact surface. WECs are networks of microcracks with an associated microstructural alteration called white etching area (WEA) which borders or is intermixed with the WEC. The appearance of WEA is revealed when etched in nital solution (2% nitric acid in ethanol). WEA is a nanocrystalline ferrite structure of grain sizes ~ 5–300 nm, ~ 10–50% harder than the surrounding matrix and comprised of wholly or partially dissolved spherical carbides found to be part of the WEA formation process [3–13]. Amorphous-like phases have also been shown to be present in WEA, forming first before WEA is generated [8, 14, 15]. WEA has been proposed to exist in two ways: deformed WEA consisting predominantly of nanocrystallites and transformed WEA consisting of co-existence between nanocrystallites and amorphous phase [16].

The formation drivers as well as the initiation and propagation mechanisms for WSF and WEC in rolling bearings are still highly debated but are thought be driven by combinations of mechanical, tribochemical and electrical effects including: (1) transient operating conditions such as wind gusts, load reversals, grid engagement, braking, generating high impact loads, vibrations and slip; (2) electrothermal and electrical effects; (3) tensile hoop stresses; and (4) hydrogen release and diffusion into the bearing steel (sourced from the lubricating oil and additives or water contamination). The proposed initiation and propagation mechanisms for WSF/WECs are: (1) surface initiation through two opposing mechanisms, (1) shear stress-induced fatigue microcracks [17] and (2) localised high circumferential tensile stress spontaneously induced cleavage-like axial cracks that initiate independently [5, 17, 18], at defects such as inclusions [17–21] or due to corrosion, machining defects or electrical erosion pits [21]; (2) subsurface initiation by non-metallic inclusions (NMIs) [5–7, 22–26], perhaps in some cases due to tensile stresses [27]; (3) adiabatic shear banding independent or including defects through impact events, cracks forming after microstructural changes occur [2, 28]; (4) self-charging of lubricants triggering localised transient current flow causing local electromagnetic induction that crosses the contact surface leading to electrothermal mechanisms triggering subsequent WEA microstructural change [29, 30]; (5) a multistage initiation of WECs as a result of migration of carbon under shear stress and high localised energy [31].

Subsurface-initiated cracks are difficult to identify and quantify. When a ‘young’ crack is found, the mechanism of formation is easier to understand and is frequently believed to be revolved around NMIs. One technique used for recording WECs is the application of serial sectioning to map entire WECs in 3D. This technique has been perfected by the authors [6, 7, 24, 25] where it is confirmed that at least one mechanism of WEC formation is initiation and propagation in the subsurface, with strong evidence for subsurface initiation being at NMIs. Supporting evidence for subsurface initiation has been provided from 3D-mapping of entire WECs by X-ray microtomography conducted on high-speed wind turbine gearbox bearings (WTGBs) returned from the field [26, 32, 33] and an inner ring section of a large spherical roller bearing used in an industrial application [26]. X-ray tomography of this spherical roller bearing revealed large (> 26 μm in axial and circumferential length) multiphase inclusion combinations of Al, Mn and S elongated in the direction of over-rolling to have initiated subsurface WECs. Through metallographic analysis of field-returned WTGBs, it has also been found that small/short sized 8–24 μm MnS inclusions were mainly associated with butterfly/small-WEC crack initiation [22]. Analysis of failed low-speed shaft WTGBs, however, has found that oxide and dual phase inclusions are more detrimental than MnS inclusions [34]. Subsurface WECs and inclusion interactions have also been found through testing and metallographic analysis of WTGBs with induced tensile stresses from bearing seat deviation [27]. This supports evidence from previous studies conducted on WTGBs by authors of this manuscript [7] where predominantly small/short sized (3–20 μm) sulphides, globular oxides and globular MnS-oxide inclusions were recorded and judged likely initiators of WECs. It is proposed that small NMI-initiated WECs coalesce to form larger networks that eventually branch to the contact surface causing WSF or axial cracking [1, 4–8, 23–25].

There is also debate on whether the crack or WEA microstructural change occurs first, whether WEAs form cooperatively with the crack and whether WEA forms gradually or suddenly, where proposed formation mechanisms include amorphisation [8, 14–16, 35], adiabatic shear, severe localised plastic deformation, low-temperature recrystallisation, carbide break-up and dissolution and electrothermal effects, these being extensively reviewed in [1]. One popular hypothesis is due to crack face rubbing causing a localised mechanical deformation during RCF (this being enhanced in the presence of diffusible hydrogen [36], higher concentrations could exist at these sites [37]), an associated material transfer from one side of the crack to the other occurs, and recrystallisation results [13, 37, 38]. A more recent hypothesis developed through modelling is energy dissipation at rubbing crack faces [35]. The developed crack model quantifies the amount of energy dissipated as a result of friction at crack faces; part of this energy is converted to heat and microstructural decay, WEA formation being a result of amorphisation due to high density dislocation accumulation. The energy generated during crack rubbing leading to amorphisation is also proposed to be sufficient to dissolve large amounts of carbides in the WEA [16]. A counter argument to crack rubbing comes through subsurface inspection using Barkhausen noise measurements, where subsurface changes are investigated without the presence of cracks [39]. A local transformation in the microstructure is observed as ‘crack-free’ irregular dark etching regions and is suggested to lead to the formation of WEAs. Similarities have been shown between microstructural alterations in WECs, and those alterations found in dark etch regions [40]. An experimental approach by artificially inducing microcracks into the steel prior to RCF has also shown that hard WEAs formed in close proximity to the microcracks, providing an experimental validation that cracks can be a precursor to WEA formations [12]. In an investigation to study the effect of brittleness on the generation of WEA, modified AISI 8620 steel was intentionally heat treated to produce intergranular embrittlement [41]. During RCF, cracks formed preferentially along the grain boundaries due to lowered toughness where WEA was found to form along these intergranular cracks. It is suggested that the movement of the crack faces under subsurface shear promoted the formation of WEAs along the crack faces. Finally subsurface crack rubbing has been shown to produce wear debris with an identical composition to the steel matrix, the wear debris being a result of the disintegration of lamellar structure formed during crack rubbing [16].

This study uses extensive metallographic analysis including standard and serial sectioning techniques to record and map individual WECs in 3D and associated damage features in RCF-tested 100Cr6 steel cylindrical roller thrust bearings (CRTBs) on an FAG-FE8 test rig, this being a continuation of the works conducted previously by the authors [42, 43]. Many previous studies have applied different techniques to record and map WECs in failed bearings from the field and RCF-tested samples; however, little attempt has been made to record the evolution of WECs from their initiation to final flaking. This study aims to provide evidence for the stages of subsurface inclusion initiated WEC evolution for the first time to give valuable insight into this bearing degradation mechanism.

There is an accompanying piece of work [44] to this investigation that explores the role of hydrogen diffusion in the RCF tests conducted in this study.

## Materials, Techniques and Experimental Methods

### Rolling Contact Fatigue Testing

**Fig. 1:**
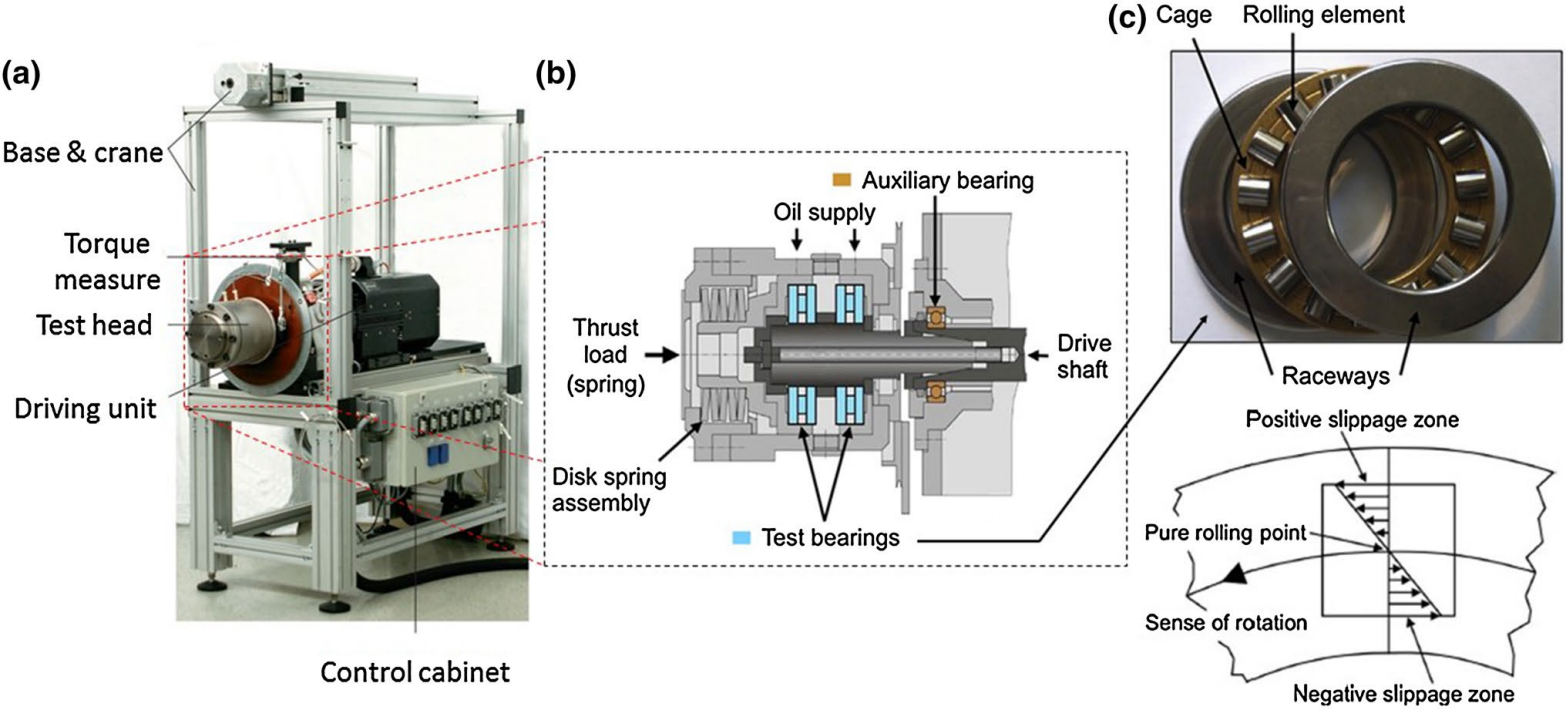
**a** FAG-FE8 test rig, **b** schematic of test chamber (side on view), and **c** CRTB used in the RCF testing and slippage condition experienced. Adapted from [24]

Testing was conducted on a standard FAG-FE8 rig (see Fig. 1). Two 100Cr6 steel CRTBs are tested simultaneously, usually used in the standardised test (DIN 51819-3). Two plate springs apply load. Each bearing has 15 individual rollers mounted in a brass cage sandwiched between two washer raceways (see Fig. 1c). The raceways are pre-roughened before testing to *R*_q_ values of 0.25 and 0.5 µm for the two bearings, respectively, the 0.5 µm bearing being focused upon. On the raceway at the centre of the bearing, contact zone exists a pure rolling condition with rising slip to the edges of up to ± 12.5% SRR along the contact major axis (see Fig. 1c, [45]). On the rollers, the slip zones experience both − ve and + ve directional slip, due to the roller being sandwiched between two raceways. Eight tests were conducted from 0 to 18 h, two of the tests (at 16.5 and 18 h) were shut down due to a vibration threshold limit being reached; other tests were shut down manually at pre-determined running durations (0 h control, 2 h, 4 h, 6 h, 6 h repeat and 12 h). The calculated maximum Hertzian contact pressure Pmax is in the range of ~ 1.5–1.9 GPa, this being in accordance with the contact length used for a range of lengths between 7 and 9 mm (this dependence taking into account the profile of the rolling element geometry and where roller/raceway contact is assumed to start based upon bearing drawings and software). The test conditions are shown in Table 1.

**Table 1:** FAG-FE8 RCF test conditions

*Test system*	
Test rig	FAG-FE8
Test sample	Cylindrical roller thrust bearings
Bearing type	F-562831-01/81212
Oil properties	
Oil type	Automotive gear oil, fully formulated semi-synthetic (ISO VG64)
Viscosity	64 cSt (40 °C), 9.5 cSt (100 °C)
Pressure viscosity coefficient (α)	6.6 GPa^−1^
Dynamic viscosity η_o_ (100 °C)	0.0046 Pas
Oil additives	Sodium and calcium anti-corrosion sulphonates, ZDDP antiwear additives, VI improvers and friction modifiers
*Bearing material properties*	
Washer/roller/cage material	Martensitic 100Cr6 steel/martensitic 100Cr6 steel/Brass
Hardness roller/washer	765 HV/590 HV
Surface roughness (R_q_) roller/washer	0.09 μm/0.50 μm
*Test conditions*	
Rotational shaft speed	750 rpm
Axial load	60 kN
Max contact pressure	~ 1.5–1.9 GPa (depending on contact length used between 7 and 9 mm, 9 mm used in this study)
Bearing/oil temperature	100 °C
Minimum film thickness (*h*_min_)	0.053 μm
Lambda ratio	0.1
*Test durations*	
Test number (duration) 1/2/3/4/5/6/7/8	0/2/4/6/6-repeat/12/16.5/18 (h)
Subsurface shear stresses	
Max orthogonal shear stress (*τ*_o, max_)	~375–475 MPa (acting at a depth below the contact surface of ~ 92 μm)
Max unidirectional shear stress (*τ*_uni, max_)	~ 456–578 MPa (acting at a depth below the contact surface of ~ 145 μm)

Initial minimum oil film thickness (*h*_min_) between rollers and washer raceways is calculated using the Hamrock and Dowson visco-elastic equation [46, 47]; see Eq. (1). Lambda ratio (*λ*) has been calculated based upon *h*_min_ and the roughness (*R*_q_) values given in Table 1, the bearing running in boundary lubrication throughout RCF testing; see Eq. (2). Fully formulated semi-synthetic gear oil was used as the lubricant for all tests (detailed in Table 1). The oil temperature in the contact is controlled at 100 °C during operation.

It should be noted that no method to artificially induce WECs was used, such as pressure transients, hydrogen charging or applying electrical currents. The pressure, speed and temperature are in steady state. The lubricant used is known to readily induce WSF.

### Metallographic Analysis and Contact Surface Inspection

Metallographic analysis including fine and coarse serial sectioning techniques was conducted on the RCF-tested bearing rollers and raceway washers to record and fully map individual WECs in 3D and associated damage features. Optical macroscopy was used to inspect the contact surface of the bearing parts before metallographic analysis.

**Fig. 2:**
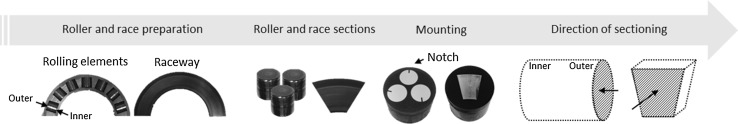
Stages of sample preparation for rollers and raceway sections

Before sectioning, the raceway washers were cut into ~ 20 × 20 mm sections, the rollers kept whole. Rollers and raceway sections were subsequently hot mounted in Bakelite. Rollers were mounted in groups of three for each test duration (excluding the 16.5-h test) to make sure analyses were statistically representative of each test duration. Raceway sections for the 18-h test only were mounted singularly, four individual sections being analysed covering ~ 1/3rd of the washer to inspect a representative area of steel. Notches were cut into the rollers to act as a marker for identifying individual WECs at each sectioning slice interval around the circumference, individual WECs, when recorded, being numbered in regard to the numbers around a clock face, the notch representing 12 o’clock. Rollers were mounted such that they were sectioned in the axial direction from the outer roller edge through to the inner. Raceway washers were mounted such that the contact surface was sectioned, material removal being performed in the radial z-direction. See Fig. 2 for sample preparation.

Sectioning was performed on automatic grinding/polishing machines (Struers TegraPol-15 with a TegraForce-1 and a Struers LaboPol-21 with a LaboForce-3) using 800, 1200 and 4000 SiC papers followed by 9 and 3 µm diamond suspension lubricants for the final polishing stages. The polished sample cross sections were then chemically etched in nital (2%) before imaging. Images were taken by optical microscopy (Olympus BX41 M-LED and BX51) at 50–200× magnification to map and record WECs, with 500× and 1000× magnifications accompanied with SEM/EDX (JEOL JSM-6500F SEM and Oxford Inca 300) to image and analyse inclusion–WEC interactions. Open source image processing software (ImageJ) was used to measure the dimensions of WECs and damage features. To create a controlled sectioning process, macro-Vickers indents were used to track the grinding/polishing removal rate as well as being used to track individual WECs.

#### Contact Surface Inspection

Optical macroscopy was conducted on the same rollers and raceway sections that were subsequently sectioned. Images were taken at 60° intervals around the circumference of the rollers. The four individual raceway sections were imaged to give an overview of the contact surface.

#### Sectioning Analysis

Fine serial sectioning was conducted on the 2-, 6- and 18-h RCF tests with removal intervals of ~ 3.4–3.9 µm per slice.

Coarse serial sectioning was conducted on the 4- and 12-h tests, and after fine serial sectioning on the same 6- and 18-h test rollers to continue recording WECs across the length of the rollers. Coarse serial sectioning was not conducted on the 2-h test due to no WECs being found through fine serial sectioning. Removal intervals were conducted at ~ 15, 30 or 50 µm per slice. Coarse serial sectioning was conducted from the outer roller edge through to the inner on the 6-, 12- and 18-h tests, the 4-h test being cut short due to lack of WECs recorded. Coarse serial sectioning was conducted up to ~ 9.6–10.41 mm across the 6-, 12- and 18-h tests, this being due to time considerations and that through initial macrosectioning WECs were first found at ~ 1.7–2.6 mm from the outer edge. Inclusions were recorded during coarse serial sectioning; only inclusions recorded in the outer roller half being displayed. Coarse serial sectioning was conducted on the 18-h raceway sections at intervals of ~ 50 µm, starting at the contact surface (0.00 mm) up to a total depth of ~ 500 µm. The individual fine and coarse serial sectioning interval ranges for rollers and raceway sections are listed in Table 2.

**Table 2:** Sectioning intervals and removal rates conducted during the metallographic analysis

Test duration	Analysis type	No samples analysed	Sectioning interval (~ mm)			Slice removal rate (~ μm)	
			Macrosectioning	Fine serial	Coarse serial	Fine serial	Coarse serial
2 h	Fine serial	3× Rollers	R1: 0.00–1.65	R1: 1.65–2.22	–	R1: 3.4	–
			R2: 0.00–1.76	R2: 1.76–2.37	–	R2: 3.6	–
			R3: 0.00–1.51	R3: 1.51–2.17	–	R3: 3.9	–
4 h	Coarse serial	3× Rollers	R1: 0.00	–	R1: 1.7–2.84	–	R1: 15
			R2: 0.00	–	R2: 1.86–2.8	–	R2: 15
			R3: 0.00	–	R3: 1.7–2.82	–	R3: 15
6 h	Fine + coarse serial	3× Rollers*	R1: 0.00–1.92	R1: 1.92–2.68	R1^+^: 2.68–10.3	R1: 3.4	R1^†^: 15/30/50
			R2: 0.00–1.73	R2: 1.73–2.57	R2^+^: 2.57–9.6	R2: 3.7	R2^†^: 15/30/50
			R3: 0.00–1.51	R3: 1.51–2.28	R3^+^: 2.28–9.67	R3: 3.4	R3†: 15/30/50
12 h	Coarse serial	3× Rollers	R1: 0.00	–	R1: 1.8–10.26	–	R1^†^: 15/30/50
			R2: 0.00	–	R2: 2.03–10.41	–	R2^†^: 15/30/50
			R3: 0.00	–	R3: 1.86–9.7	–	R3^†^: 15/30/50
18 h	Fine + coarse serial	3× Rollers* 4× Raceways	R1: 0.00–2.01	R1: 2.01–2.59	R1^+^: 2.59–10.27		R1^†^: 15/30/50
			R2: 0.00–1.77	R2: 1.77–2.41	R2^+^: 2.41–9.8		R2^†^: 15/30/50
			R3: 0.00–1.77	R3: 1.77–2.36	R3^+^: 2.36–9.64	R1: 3.4	R3^†^: 15/30/50
					RW1: 0.00–0.047	R2: 3.8	RW1: 50
					RW2: 0.00–0.051	R3: 3.5	RW2: 50
					RW3: 0.00–0.048		RW3: 50
					RW4: 0.00–0.047		RW4: 50

All samples are macrosectioned at ~ 50 μm slice intervals at the start outer rolling element edge (0.00 mm) until the first visible sign of WECs are found in any one test roller. R denotes rolling element, and RW denotes raceway washer

*Same samples used for fine and coarse serial sectioning analysis, samples mounted as a group of 3

^+^Coarse serial sectioning conducted after fine serial sectioning analysis

^†^Slice intervals of ~ 15 μm conducted initially, with intervals of ~ 30 and 50 μm being conducted subsequently due to time considerations

#### WEC Tomography and 3D Crack Modelling

One of the individual WECs recorded in the 6-h test (WEC-10 R2) had each fine serial sectioning slice interval image at 200× magnification aligned using layering tools using Photoshop CS3 and subsequently stacked. The image stacks are then used to make a video using Fiji software showing an orthoslice sweep through the volume of steel showing the WECs morphology in 3D from start to finish.

The total WEC damage recorded in one individual roller (Roller 1) from the 18-h test was modelled in 3D. Optical microscope images at 200× of every individually recorded WEC in the roller were segmented at 0.25 mm intervals across the roller from outer to inner edge (0–11 mm). These images were then aligned and stacked before being imported into Fiji software where interpolation between individually segmented WECs was conducted. 3D models were subsequently constructed using Aviso and VGStudio MAX software. Animations of the 3D model were constructed using VGStudio MAX and Fiji.

#### WEA Volume Analysis

**Fig. 3:**
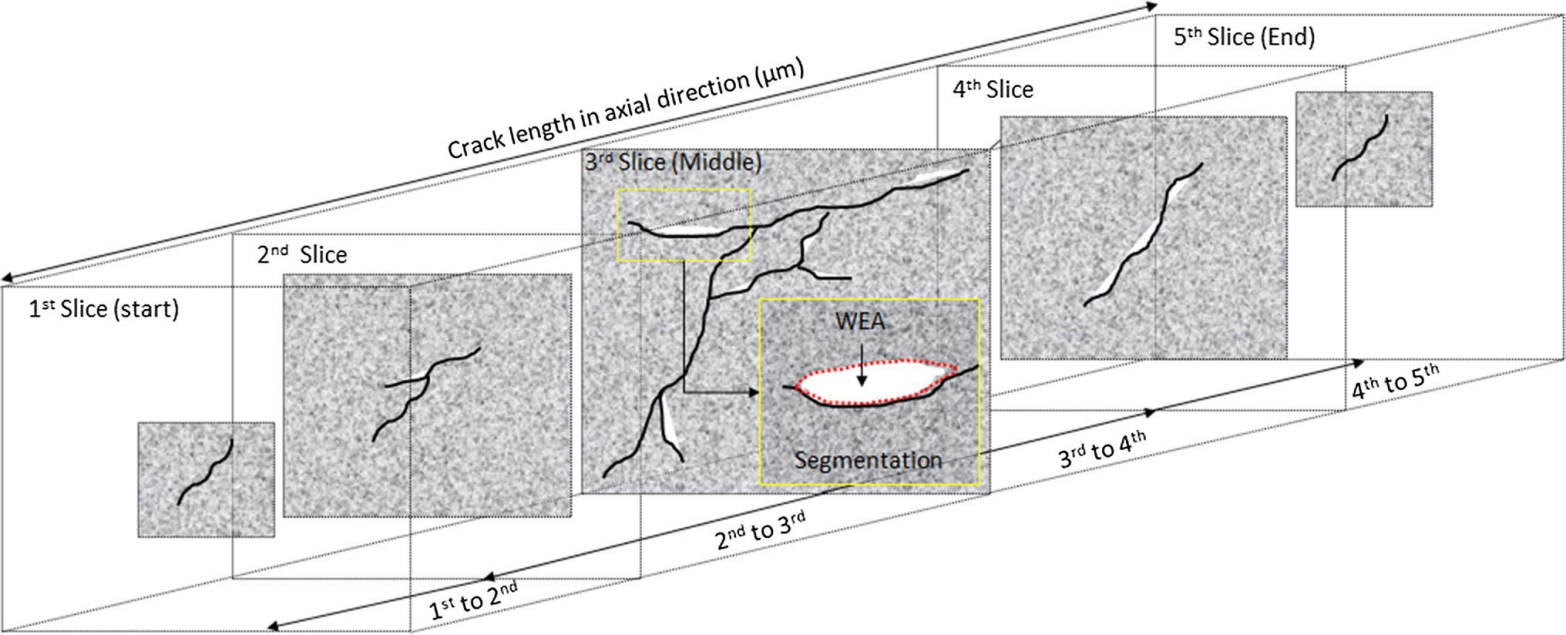
Schematic illustrating the methodology of WEA volume measurements

ImageJ has been used to quantitatively analyse the average total volume (μm^3^) and area (μm^2^) of white etching microstructural alteration associated with individual WECs across the RCF test durations. Five individual slices taken from start to finish across four individual WECs from the 4–18-h tests were analysed (see Fig. 3). At each slice (1–5), the WEA associated with the WEC is segmented in 2D to give the total WEA (μm^2^) for that particular slice. Multiplication of the individual total WEAs at the 2nd, 3rd (middle) and 4th slices with the axial length between slices plus the total WEA at the 1st and 5th slices gives the total WEA volume (μm^3^) for that particular WEC, i.e. 1st WEA + 2nd WEA (axial distance between 1st and 2nd) + 3rd WEA (axial distance between 2nd and 3rd) + 3rd WEA (axial distance between 3rd and 4th) + 4th WEA (axial distance between 4th and 5th) + 5th WEA. The average WEA volume for each RCF test duration is then calculated for comparison based upon the 4 WECs analysed. It is important to note that this approximates the WEA volume found associated with the crack, and therefore measurements can be over-/underestimated and not fully representative of the actual amount of WEA present.

**Fig. 4:**
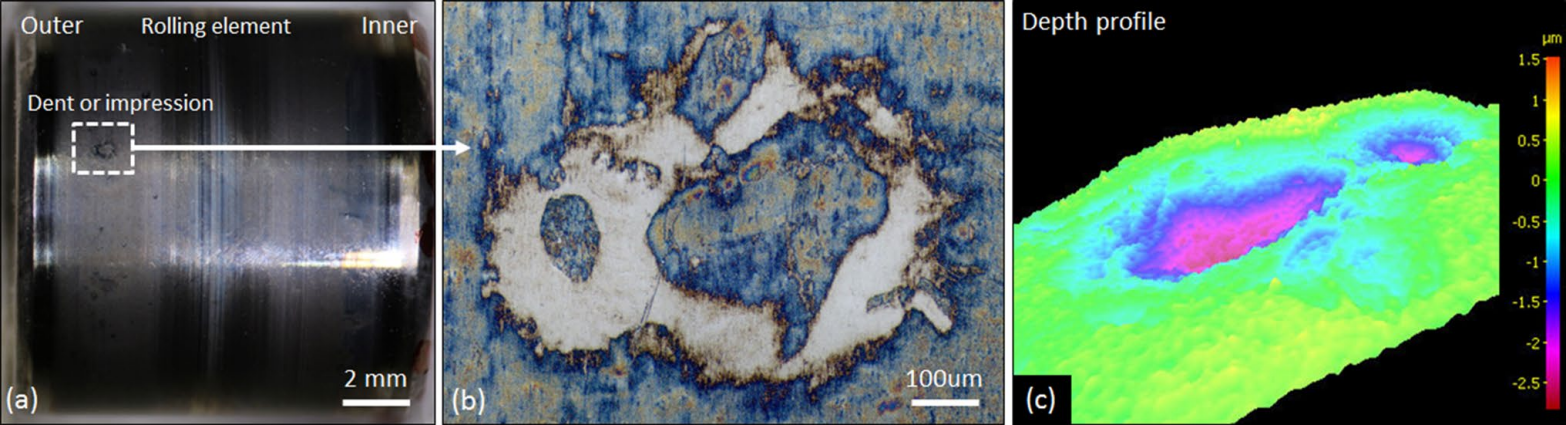
Surface analysis of a typical indent found on the 18-h rollers. **a** Optical macroimage of roller indicating location of indent on the contact surface. **b** Magnified optical image of the indent. **c** Depth profile analysis of the indent

**Fig. 5:**
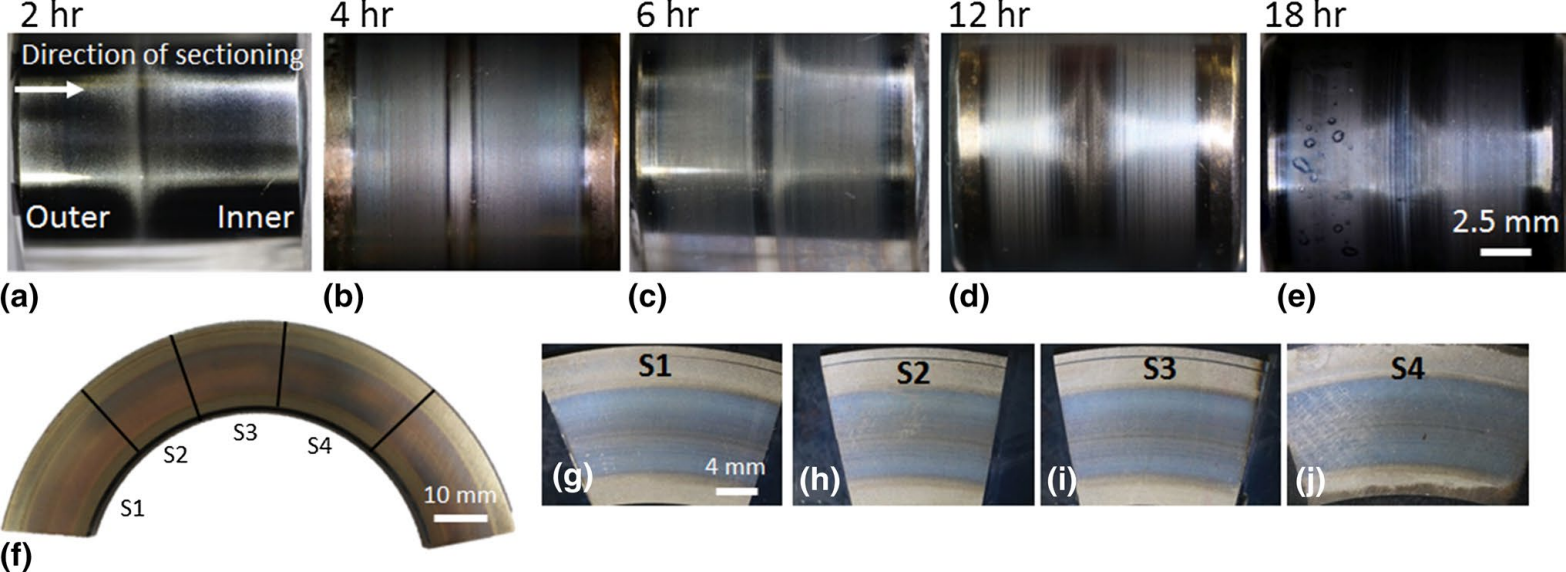
Optical macroscope images of a single roller from each RCF test duration (2–18 h) and 18-h raceway sections chosen for subsequent sectioning. Images **a**–**e** show one of the 60° interval zones around the circumference of the roller. **f** Overview image of raceway sections (S1–S4). Images **g**–**j** are optical macroimages of the corresponding sections (S1–S4) shown in **f**. S denotes sample number

**Fig. 6:**
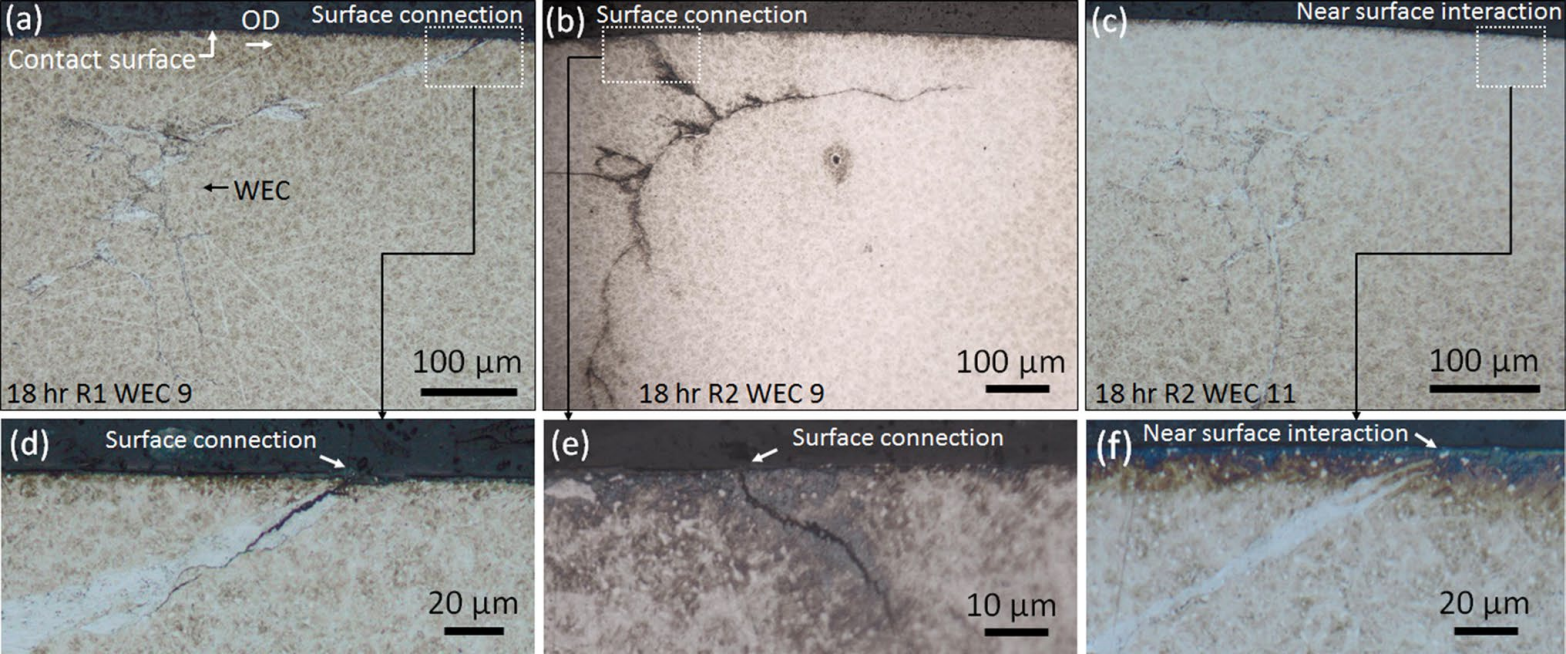
Optical images of 18-h surface connections and near-surface interactions. **a** WEC-9 R1 surface connection, surface connection length in axial sectioning direction < 30 μm. **b** WEC-9 R2 surface connection, surface connection length ~ 500 μm. **c** WEC-11 R2 near-surface interaction. **d**–**f** ×500 optical images of the surface connections and near-surface interactions shown in (**a**–**c**). Over-rolling direction (OD) left to right

**Fig. 7:**
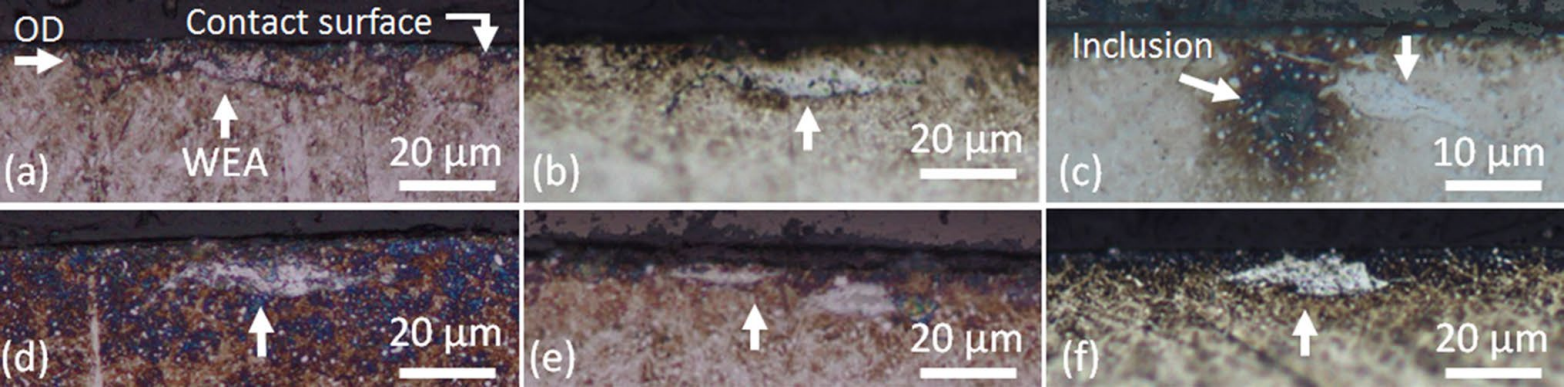
Optical images of typical 18-h near-surface WEA/WEC features (**a**–**f**). Over-rolling direction (OD) left to right

**Fig. 8:**
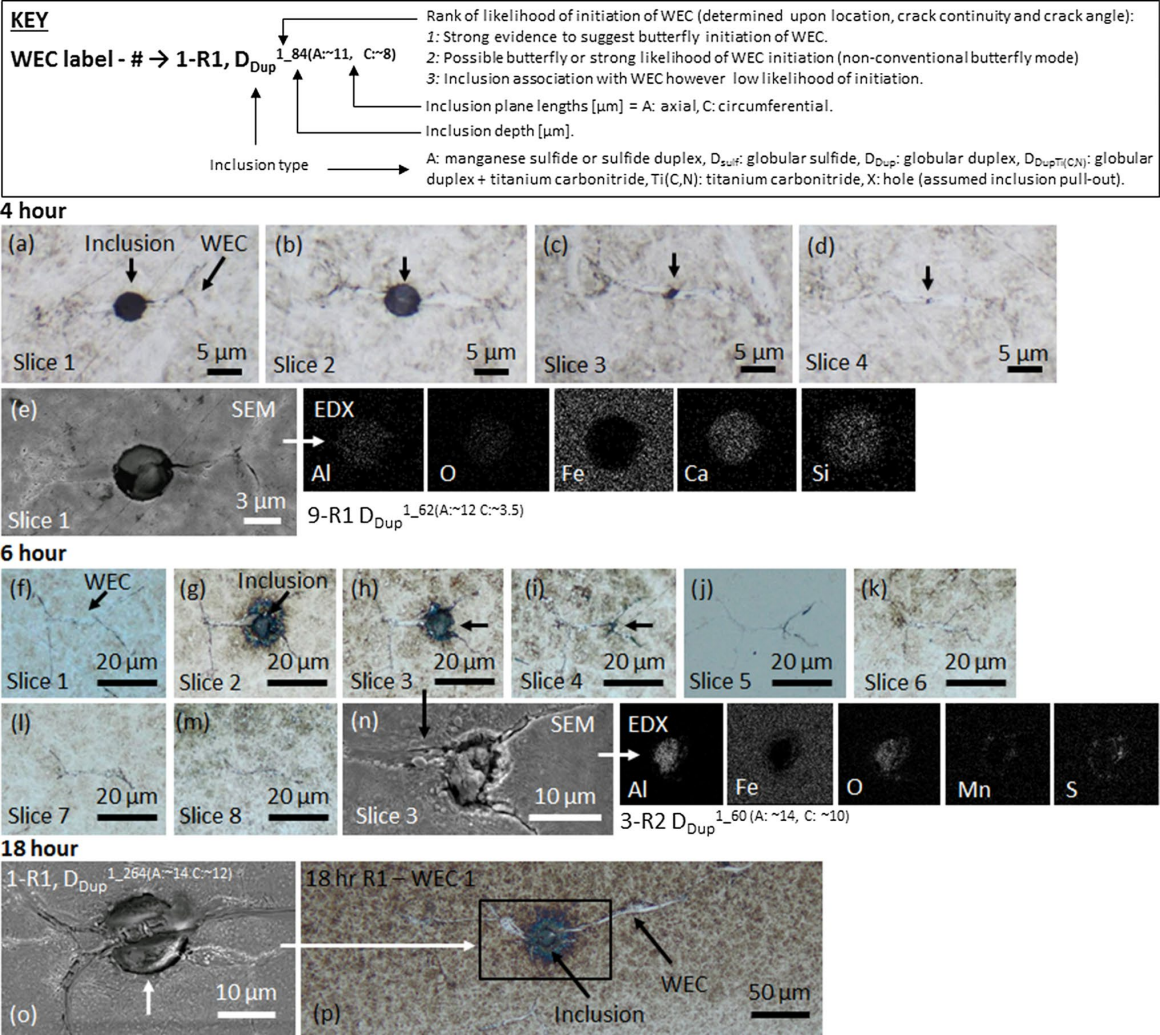
Optical images of mapped WECs and inclusion–WEC interactions at 4, 6 and 18 h. **a**–**d** represent the individual slices from start (slice 1) to finish (slice 4) of mapped 4-h WEC-9 R1. SEM image, **e** shows inclusion–WEC interaction from (a) slice 1 with corresponding EDX chemical maps. **f**–**m**) Mapped 6-h WEC-3 R2, images (**f**–**m**) represent the individual slice images from start (slice 1) to near finish (slice 8). Images **g**–**i** show the location of the inclusion–WEC interaction. **n** is an SEM image of the inclusion–WEC interaction from **h** (slice 3) with corresponding EDX chemical maps. **o** SEM image of inclusion–WEC interaction recorded in 18-h WEC-1 R1 with corresponding location of the inclusion–WEC interaction shown in optical image **p**. A key above the images details how to interpret the inclusion–WEC interaction information in each case

**Fig. 9:**
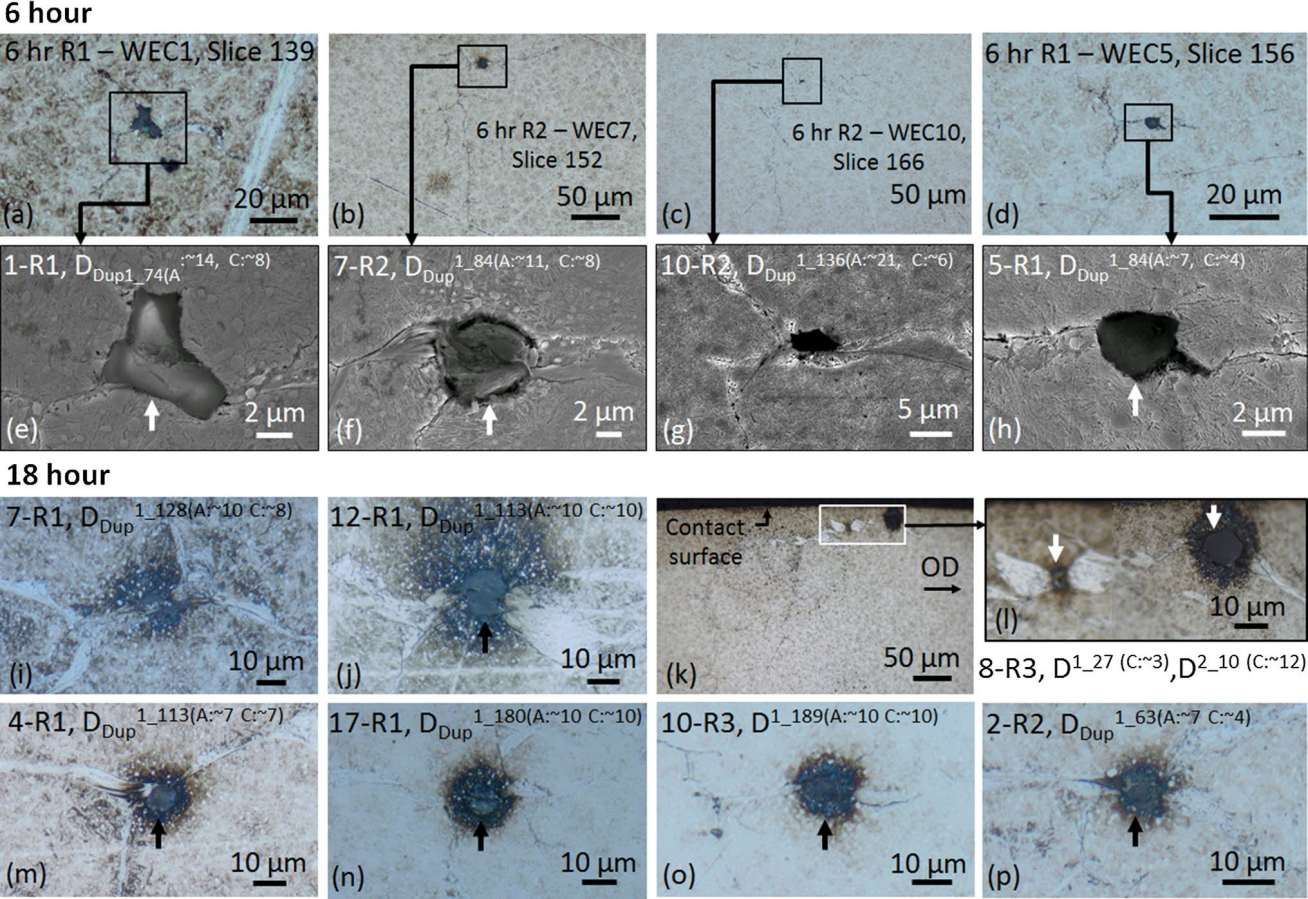
Images of typical inclusion–WEC interactions recorded at 6 and 18 h. Images **a**–**d** show the location of the inclusion–WEC interactions from SEM images **e**–**h,** respectively, at 6 h. Images **i**–**p** are optical images of typical inclusion–WEC interactions recorded at 18 h. **k** and **l** show an example of a butterfly WEC with corresponding inclusion linking to another inclusion in the WEC network, **l** showing a magnified image of the highlighted region. Arrows highlight the inclusion in each case. See Fig. 8 and ‘Appendix B’ for more information on the inclusion ranking system

**Fig. 10:**
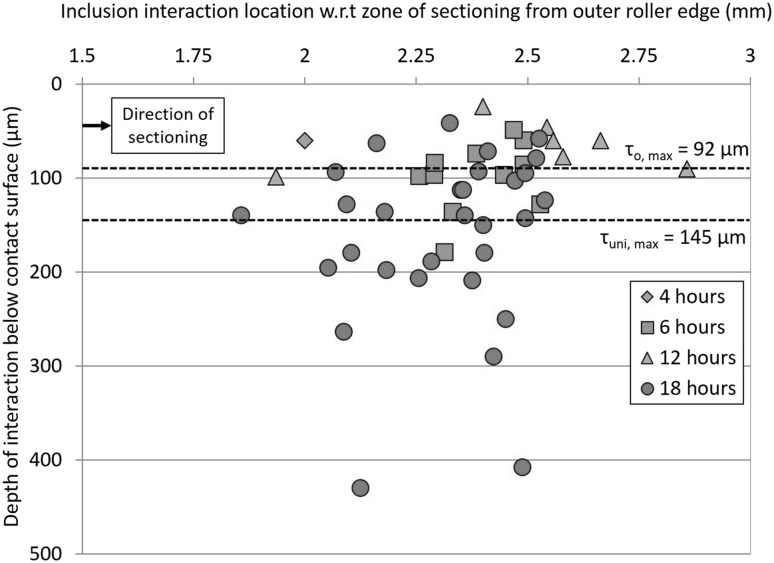
**a** Spatial distribution and depth of inclusion–WEC interactions w.r.t the depth of maximum subsurface sheer stresses judged to have a high likelihood of crack initiation (rank 1 or 2). See Fig. 8 and ‘Appendix B’ for more information on the inclusion ranking system

**Fig. 11:**
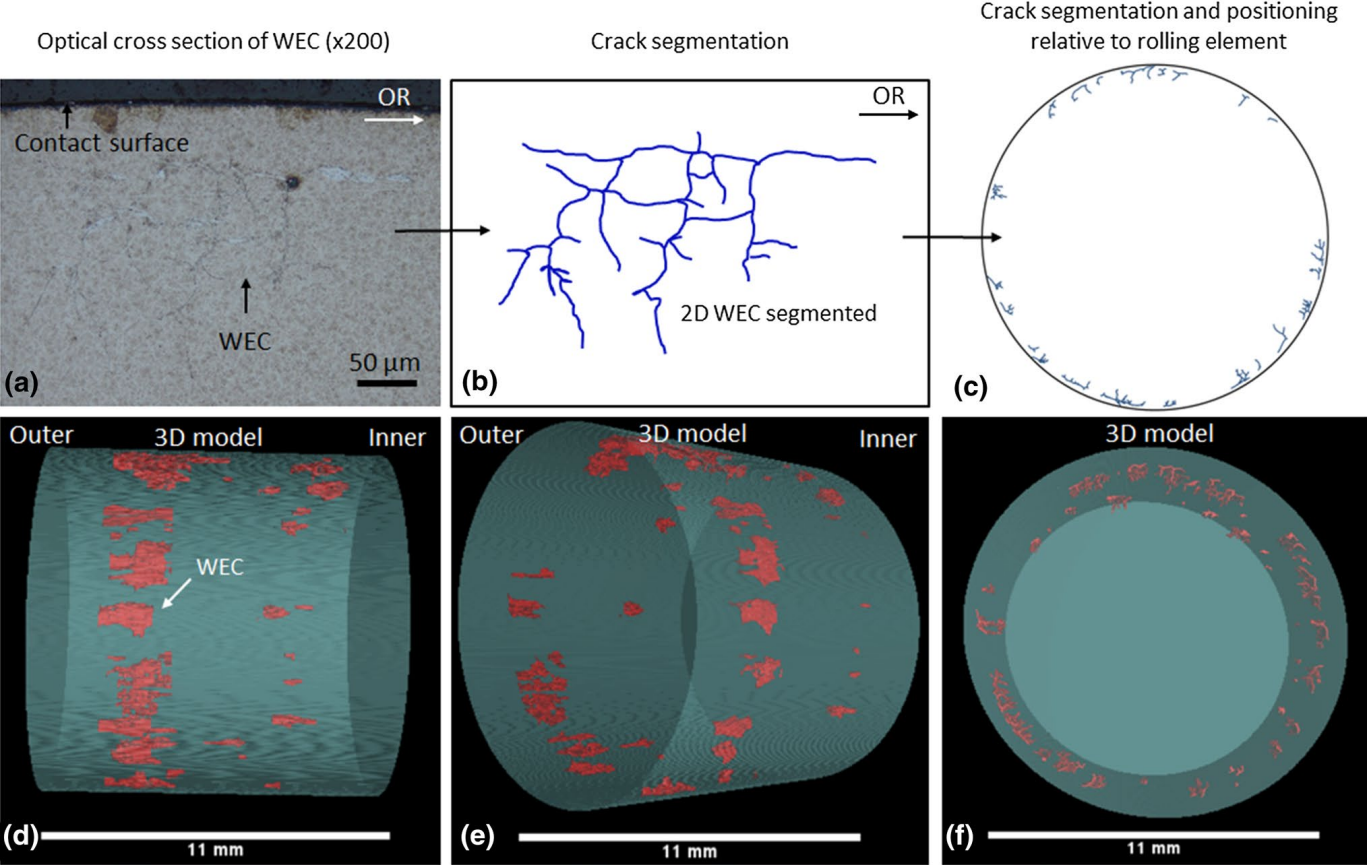
3D model of total WEC damage recorded across 18-h R1. **a** Optical cross-sectional image of a WEC. **b** 2D segmentation of WEC from optical image in (**a**). **c** Placement of 2D segmented WEC into its relative position across the roller. **d**–**f** 3D model with all WECs highlighted in red across the roller from outer to inner edge. See Video 2

**Fig. 12:**
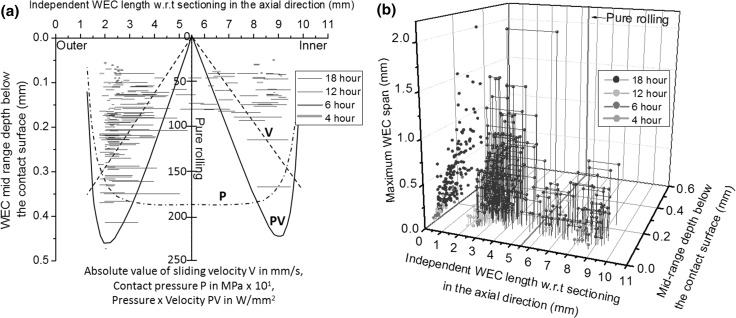
**a** Distribution of individual WECs recorded in 4–18-h rollers across the entire axial length (x-axis) of rollers from outer to inner edge and corresponding mid-range depth below the contact surface (0.00 mm) (y-axis). Pressure P, absolute sliding velocity V and slip energy PV are also represented (adapted from [49]). **b** 3D plot of all independently recorded WECs across 4–18 h. X-axis represents the entire axial length of the roller from outer to inner edge. Y-axis represents the mid-range depth below the contact surface (0.00 mm). Z-axis represents the maximum span (see ‘Appendix C’ for details). The dots on the YZ-projection represent the position of each independent WEC in the Y-axis and Z-axis. The distances between spheres for each WEC represent the total length in the X-axis

**Fig. 13:**
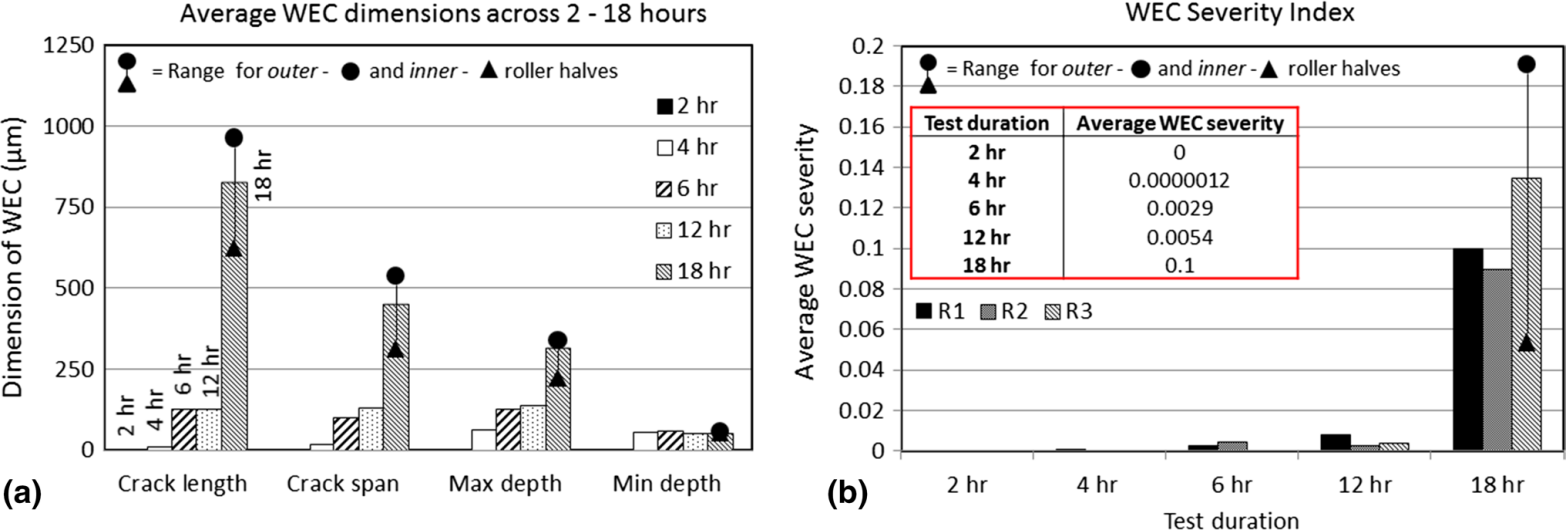
**a** Average WEC dimensions in the rollers across 2–18-h tests, see ‘Appendix C’ for details on measurement of relevant crack dimensions. **b** WEC severity index in the rollers for the 2–18-h tests

**Fig. 14:**
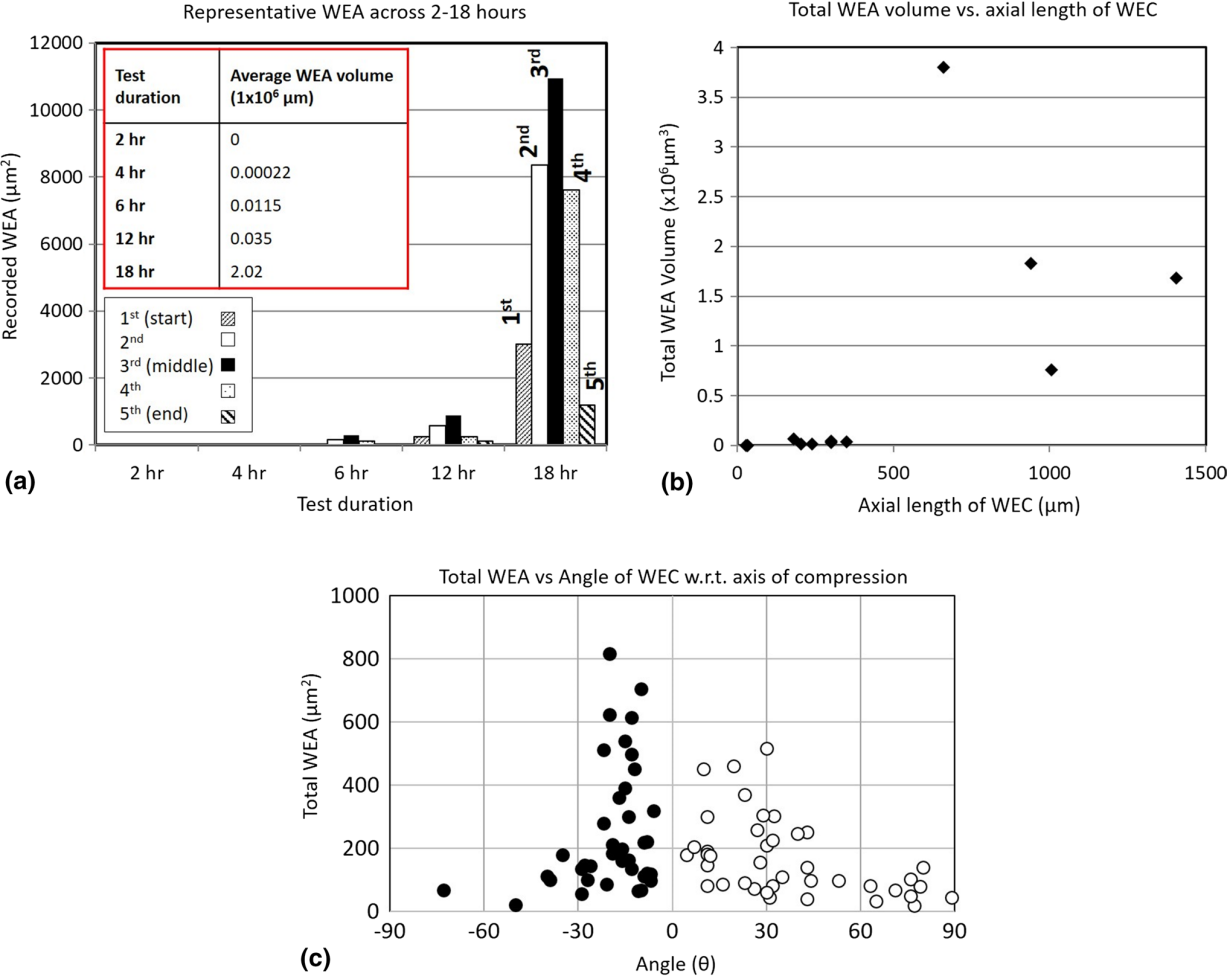
WEA volume and area analysis. **a** Representative average WEA across 2–18 h. **b** Total WEA volume versus axial length of WEC. **c** Total WEA measured vs the respective angle of the WEC for 5 different randomly chosen WECs at 18 h

**Fig. 15:**
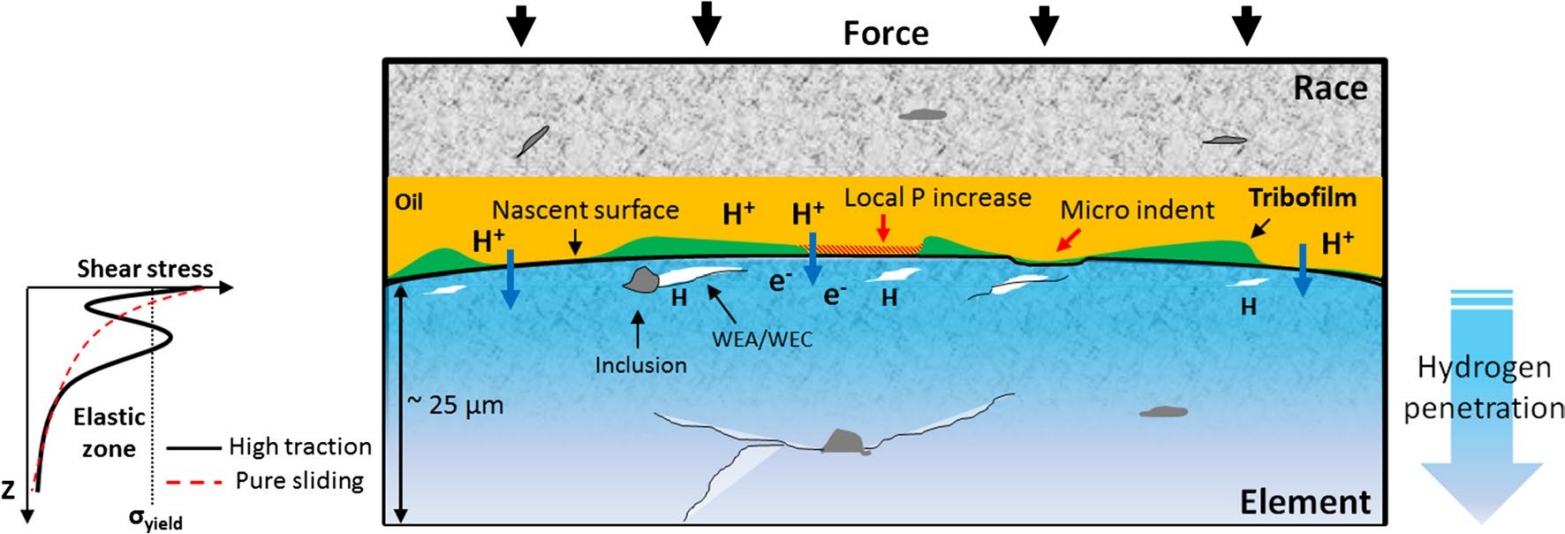
Hypothesised mechanisms of near-surface (< 25 μm) WEA/WECs formation. H^+^ denotes molecular hydrogen, H denoting monoatomic hydrogen diffused into the bearing steel. e^−^ denotes free electrons at the fresh nascent surface. ‘P’ and ‘σ’ denote pressure and yield stress, respectively

**Fig. 16:**
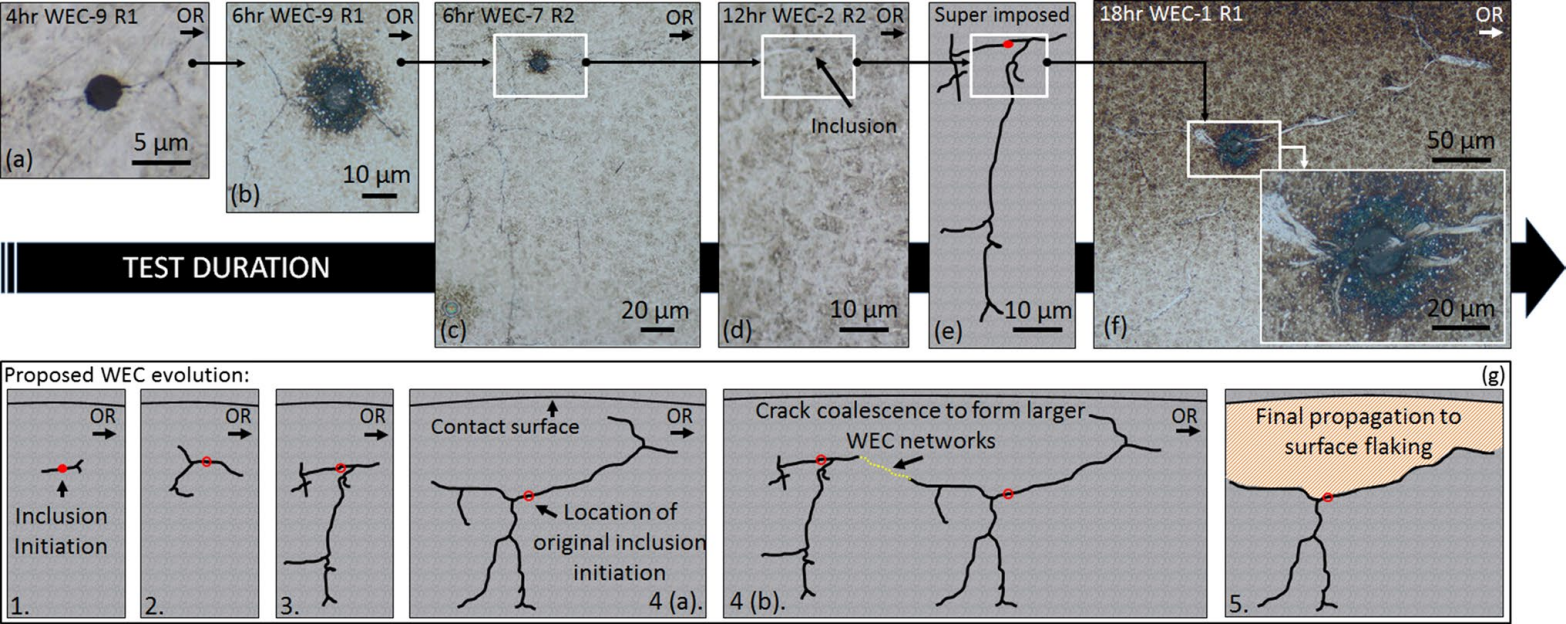
Optical images of WECs recorded at 4–18 h. **a**–**f**) show examples of typical WECs recorded between 4 and 18 h. **e** Super-imposed image of the 12-h WEC shown in (**d**), the inclusion highlighted in red. **g** Proposed stages of WEC evolution; (1) initiation via inclusion in the subsurface, (2) propagation into ‘star-like’ WEC (see b), (3) further propagation in radial and over-rolling direction, (4a) continued propagation, (4b) coalescing of independent WECs to form larger WEC networks and (5) final propagation to the surface resulting in flaking. Over-rolling direction (OR) is from left to right (Color figure online)

**Fig. 17:**
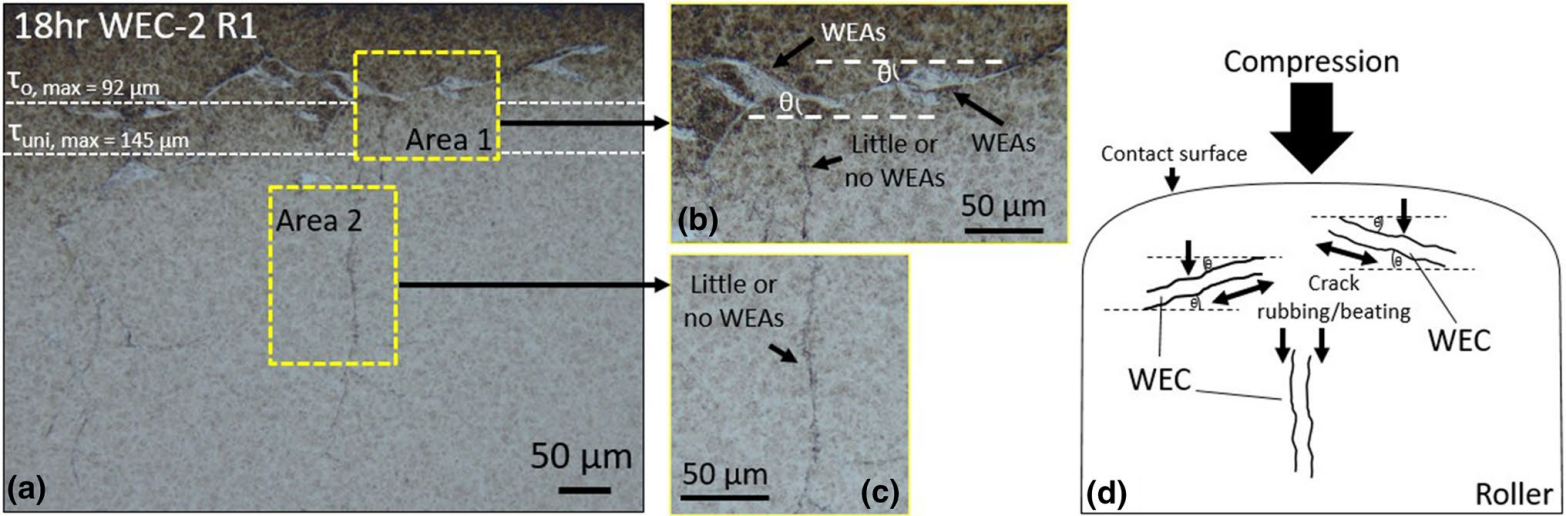
Optical image of a WEC demonstrating the influence of crack angle w.r.t the axis of compression and depth of maximum subsurface shear stresses (τ_0, max_ = 92 μm, τ_uni, max_ = 145 μm) on the formation of WEAs. **a** Areas 1 and 2 show two different orientations of crack propagation, Area 1 shows cracks at an angle θ to the axis of compression along with a vertical crack parallel to the compression axis, and the zones of maximum subsurface shear stresses are also shown. Area 2 shows a vertical crack parallel to the compression axis. **b**, **c** show magnified ×500 images of the two areas, respectively. **d** is a schematic demonstrating the influence of crack orientation angle w.r.t the direction of compression and the associated action of crack rubbing/beating

In addition, ImageJ has also been used to quantify the area (μm^2^) vs. WEC angle w.r.t the axis of compression (*θ*) (see Fig. 17d) for 5 individual WECs from the 18-h test. For each WEC, one plane from the sectioning analysis has been taken (approximately the middle slice, see Fig. 3). Individual WEAs have then been segmented out in the same way as described above and in Fig. 3. However, in addition the angle of the WEC w.r.t the axis of compression associated with the segmented WEA region is recorded. Cracks are recorded between 0° (a crack that is perpendicular to the compression) and ± 90° (a vertical crack in the same direction as the compression).

### Materials Characterisation

#### Steel Cleanliness

Steel cleanliness analysis was performed under ISO 4967-B standard [48] to measure the density of NMIs in the rollers and raceway washers. Analysis was conducted at the Dr. Sommer Werkstofftechnik GmbH laboratory. An Olympus BX51M optical microscope and software (analysis auto version 5.2 + Inclusion Inspector) was used for automatic detection of the inclusion size, type and count. A global severity index (*C*_*i*_) was given to the inclusions recorded during the analysis. Under the thresholds set by the ISO 4967-B standard, the global cleanliness index *C*_*i*_ was found to be 0.2 and 1.5 for the raceway and roller, respectively. Analysis of the raw cleanliness data (these data including the addition of NMIs recorded outside of the thresholds set by the ISO 4967-B standard) gave global cleanliness index *C*_*i*_ of 35.6 and 344.1 for the raceway and roller, respectively.

## Results

### Contact Surface Inspection

Wear across the rollers for increasing RCF test durations has been observed on both sides of the central pure rolling region corresponding to the outer and inner zones (see Fig. 5). RCF test durations of 2–12 h showed very little surface damage. The 18-h test showed visible signs of damage on the outer half in the form of dents/impressions, but conversely little surface damage on the inner (see Figs. 4a, 5). Surface analysis of a typical indent using SEM (JEOL JSM-6500F) and optical profilometry (Alicona InfiniteFocusSL) revealed the indent to have a width of ~ 800 μm and maximum depth of ~ 2 μm (see Fig. 4). No signs of spalling were found on the rollers that were subsequently sectioned; however, spalling was observed on other rollers from the same bearing. Figure 5 shows the wear and damage across the 2–18-h rollers. Inspection of the raceway washer sections at 18 h revealed little surface damage. Again wear zones can be seen on either side of the central pure rolling region, see Fig. 5.

### Metallographic Analysis

#### Fine Serial Sectioning

The results from the fine serial sectioning analysis are described below and are detailed in Table 3.

**Table 3:** Summary of WECs and inclusion interactions recorded from combined fine and coarse serial sectioning analysis

Test duration	WECs recorded	Surface connections	Near-surface WEC/WEAs	Inclusion interactions	Butterflies	WECs recorded in roller halves	
						Outer	Inner
2 h	Total: 0	Total: 0	Total: 0	Total: 0	Total: 0	Total: 0	Total: 0
4 h	R1: 1	R1: 0	R1: 0	R1: 0	R1: 7	R1: 1	R1: 0
	R2: 0	R2: 0	R2: 0	R2: 0	R2: 2	R2: 0	R2: 0
	R3: 0	R3: 0	R3: 0	R3: 1	R3: 2	R3: 0	R3: 0
	Total: 1	Total: 0	Total: 0	Total: 1, Total: 1*	Total: 11	Total: 1	Total: 0
6 h	R1: 6	R1: 0	R1: 0	R1: 5	R1: 14	R1: 5	R1: 1
	R2: 10	R2: 0	R2: 0	R2: 8	R2: 12	R2: 7	R2: 3
	R3: 6	R3: 0	R3: 0	R3: 0	R3: 4	R3: 6	R3: 0
	Total: 22	Total: 0	Total: 0	Total: 13, Total: 13*	Total: 28	Total: 18	Total: 4
12 h	R1: 13	R1: 0	R1: 0	R1: 4	R1: 4	R1: 8	R1: 5
	R2: 9	R2: 0	R2: 0	R2: 4	R2: 4	R2: 9	R2: 0
	R3: 5	R3: 0	R3: 0	R3: 3	R3: 4	R3: 2	R3: 3
	Total: 27	Total: 0	Total: 0	Total: 11, Total: 11*	Total: 12	Total: 19	Total: 8
18 h	R1: 59	R1: 9	R1: 3730	R1: 44	R1: 8	R1: 43	R1: 16
	R2: 52	R2: 9	R2: 3653	R2: 7	R2: 5	R2: 44	R2: 8
	R3: 53	R3: 1	R3: 1316	R3: 9	R3: 5	R3: 30	R3: 23 Total: 49
	Total: 164	Total: 19	Total: 8699	Total: 60, Total: 40*	Total: 18	Total: 117	

*Total number of inclusions ranked 1 or 2

##### *2* *h*

No WECs or butterflies were recorded.

##### *6* *h*

Ten individual WECs were recorded. Eight of these were mapped from start to finish (imaging at every slice), no surface connections being recorded. Through WEC tomography, the 3D morphology of one of the fully mapped WECs (WEC-10 R2) can be seen in Video 1. Five out of eight WECs had their respective images at 500× magnification aligned in strips from start to finish, an example is shown in Fig. 8. Twenty-four butterflies were recorded. Twelve inclusion interactions were recorded (see Figs. 8, 9 for examples), 9 inclusions interacting with the 8 fully mapped WECs, with multiple inclusion–WEC interactions being recorded for single WECs. Every WEC had at least 1 inclusion–WEC interaction. Small sized (~ 2–15 μm) globular sulphide-oxides (*D*_Dup_) and globular oxide (*D*) were the most common inclusions found. All 12 inclusions were evaluated as having either a rank of 1 or 2 (see details about the ranking system in ‘Appendix B’ and Fig. 8). Spatial distributions and depth of inclusion–WEC interactions are shown in Fig. 10.

##### *18* *h*

Seventy-eight individual WECs were recorded; no WECs were mapped (see [24] for 3D mapping and orthoslice sweep videos of 18.5-h WECs in their entirety). Sixteen out of seventy-eight WECs made a surface connection or near-surface interaction (< 1 μm below the contact surface); the near-surface interactions show no apparent connection with the contact surface when viewed under optical microscope at 1000× magnification (Fig. 6c). Most surface connections at 18 h have small surface connection/interaction crack volumes (< 1 μm) with short connection lengths with respect to sectioning in the axial direction (a few slice intervals) (see Fig. 6a). This being opposed to some surface connections that span over multiple slices and hundreds of microns (see Fig. 6b). A significant number of small individual WEA/WECs were also found in the near-surface zone (< 25 μm), 8700 of these features being recorded (see Fig. 7 for examples) where evidence for the propagation or coalescence of these features was not seen. Fifty inclusion–WEC interactions were recorded, multiple inclusion interactions being found for single WECs. Small sized (~ 2–15 μm) globular sulphide-oxides (*D*_Dup_) and globular oxides (*D*) were the most frequent inclusions found to interact, thirty out of fifty inclusions being ranked as either 1 or 2 regarding initiation of WECs; examples are shown in Fig. 9. Spatial distributions and depth of inclusion interactions are presented in Fig. 10. Further inclusion–WEC interaction examples from an 18.5-h RCF test can been found in [24]. Sixteen butterflies were recorded.

#### Coarse Serial Sectioning

The results from the coarse serial sectioning analysis are described below and are detailed in Table 3.

##### *4* *h*

Through analysis of 3 × rollers only, one WEC (WEC-9 R1) was found, this WEC being comparable (but smaller in size) to the smallest WECs recorded at 6 h. It should also be noted that this WEC could be judged as a butterfly; however, due to the forking nature of the crack tip it is of the authors opinion that this is an extended butterfly or WEC. WEC-9 R1 was subsequently mapped (serial sectioned). No connection to the surface was made. After mapping, standard sectioning resumed. Individual slice images of WEC-9 R1 were aligned in a strip and are shown in Fig. 8. One inclusion–WEC interaction was recorded (see Fig. 8), a small (~ 6 μm) globular Al_2_O_3_ (*D*_Dup_) rank 1 inclusion. Spatial distribution and depth of this inclusion interaction can be seen in Fig. 10. Eleven butterflies were recorded.

##### *6* *h*

Coarse serial sectioning was conducted on the same rolling elements after serial sectioning; WECs had not finished during fine serial sectioning, cracks continuing to be recorded. The total number of WECs equals the sum of WECs not finished during fine serial sectioning plus the coarse serial sectioning. Fourteen individual WECs were recorded. No surface connections were recorded. One inclusion–WEC interactions were recorded; this is likely due to the larger sectioning intervals where inclusions may have been missed or removed during sectioning due to their typical small size (~ 2–15 μm). Six butterflies were recorded.

##### *12* *h*

Twenty-seven individual WECs were recorded with no surface connections. Eleven inclusion–WEC interactions were recorded, and 12 butterflies, inclusion–WEC interaction spatial distributions are shown in Fig. 10.

##### *18* *h*

Coarse serial sectioning was conducted after serial sectioning on the same rolling elements, WECs being recorded as in the case of the 6-h test described above. One hundred and forty-five individual WECs were recorded. Nineteen surface connections were found. Nine inclusion–WEC interactions were recorded; their respective spatial distributions are shown in Fig. 10. Likewise, inclusion–WEC interactions may have been lost due to larger sectioning intervals. Two butterflies were recorded. Also note that the number of near-surface WEA/WECs features recorded through fine serial sectioning significantly decreased in number when going from the outer to the inner roller halves.

No signs of ‘conventional’ WEC/WEAs were recorded in the raceway washers. However, some fine ‘WEC-like lines’ have been observed in 18.5-h raceway segments [24], but no extensive WECs were found.

#### Summary of Sectioning Analysis

Table 3 and Figs. 12, 13 summarise the combined fine and coarse serial sectioning results. Figure 13 provides the average WEC dimensions for the 2–18-h tests.

A WEC severity index has been calculated based upon the WEC length in the axial sectioning direction, the radial length of the WEC (maximum depth minus minimum depth) and the maximum WEC span, see Fig. 13. The average severity of WEC formations across 2–18 h (averaged across 3× rolling elements) has been calculated as well as the average severity in individual rollers and the outer and inner halves at 18 h.

The total WEC damage recorded across roller 1 from the 18-h test has been modelled in 3D. This is to visualise the extent, density and distribution of damage seen typically at the late stages of RCF before failure, see Fig. 11 (see Video 2 for a 2D segmentation, 3D orthoslice sweep through the entire volume of the roller representing each individual WEC recorded across the roller from outer to inner edge). Figure 11a shows an example of an axial slice cross-sectional image of one of the WECs recorded in 18-h R1 that was subsequently segmented in 2D and placed in its relative position in the roller (see Fig. 11b, c). Figure 11d, e, f shows three views of the 3D model.

#### WEA Volume Analysis

Results show the average amount of WEA volume increases with test duration (see Fig. 14a). The total ‘white etching area’ (μm^2^) when quantifying the WEA at five individual positions within the WEC (see Fig. 3) shows that the 3rd slice (middle zone) of the WEC network has on average the greatest amount of WEA associated (see Fig. 14a). Total WEA volume versus respective WEC axial length for the WECs analysed shows that for an axial crack length over 500 μm a significant increase in total WEA volume exists (see Fig. 14b). Analysis of 5 individual WECs has shown that a greater amount of WEA is associated with a crack that propagates nearer to 0° (perpendicular w.r.t to the axis of compression). Vertically propagating cracks nearer to 90° (parallel to the axis of compression) showing far less WEA (see Fig. 14c).

## Discussion

### RCF Testing

This study has used contact pressure *P*_max_ at 1.56 GPa to recreate WECs in CRTBs on a FAG-FE8 test rig lubricated with a ‘special’ oil known to promote WSF. It must be taken into account that the FAG-FE8 test rig has differing dynamics to those experienced by WTGBs during service, e.g. the relatively high slip experienced in the CRTBs. However, large slippage can occur during rapid acceleration/deceleration of the shaft in wind turbine gearboxes [50] and transient events causing ~ 20–110% SRR in WTGBs [51]. Simulations have revealed that moderate sliding occurs (3–10% SRR) continuously for spherical roller bearings in intermediate shaft locations of the gearbox at roller–raceway interface in the unloaded zone [51].

### Features of WECs

#### Near-Surface WEAs/WECs

Serial sectioning analysis of 18-h rollers revealed a large number of small near-surface (< 25 µm zone) WEC/WEAs (see Fig. 7). These were rarely seen with connection to the contact surface, limited interaction with inclusions, and it was also found in cases that there was no apparent presence of a crack; thus, evidence shows that a crack is unnecessary for WEAs to form in this case. These near-surface features were predominantly found in the outer half of the rollers with a decrease in their respective numbers being found across the inner half. The occurrence of these near-surface features has been documented previously by the authors in FAG-FE8 tests [24], hydrogen charged TE-74 two-roller specimens during RCF testing [6] and actual WTGBs from service [1], where rare connection to the contact surface has been found with limited cases of inclusion interaction [6].

It is hypothesised that the occurrence of these features is a result of either, or a combination of; (1) an increase in traction between contacts at later stages of RCF operation, resulting in a rise of the maximum subsurface shear stress zone towards the surface. This would be significantly increased in the case of insufficient lubrication and higher surface roughness; (2) microindentations at the contact surface causing local regions of mixed/boundary lubrication regimes increasing the traction coefficient; (3) areas of localised increase in contact pressure, and (4) increase in the concentration of mobile diffusible hydrogen over longer RCF operation [44], where locally at the near-surface, higher localised penetration and concentrations may exist and aid in the acceleration of these features. Figure 15 illustrates the hypothesised mechanisms for the formation of these features.

#### WEC Initiation and Evolution

Analysis has shown that the propensity and average size (see Fig. 13) of WECs recorded in the rollers increase with RCF test duration (see Table 3, and Fig. 12, 13) no WECs being found in the raceway. Two factors to explain this could be: (1) steel cleanliness has shown that the raceway is ‘cleaner’ than the roller (see Sect. 2.3.1); (2) a lack of hydrogen being available to accelerate WEC formations [44]; and (3) the raceway is ~ 23% softer (590 HV) than the rollers (765 HV); therefore, the raceway is less prone to cracking due to an increased toughness. The importance of high toughness steel has been highlighted in the reduction in intergranular subsurface cracking and the subsequent movement of crack faces in generating WEA [41]. It is well recognised that hydrogen affects high strength steels, where hydrogen in its atomic mobile form is able to retain its mobility [52]. Hydrogen has, however, been shown to have little influence on toughness and no effect on the hardness of 100Cr6 bearings steel [53]. To confirm the non-existence of WECs in the raceway washer, at a later date 9 randomly selected individual sliver sections of raceway were mounted such that the sections were examined in the opposite axial direction (side on instead of top down contact surface direction). Two sections at 100-um intervals were taken and examined through optical microscopy. No evidence of WECs was found.

No WECs were recorded at 2 h; this could be thought logical due to the short RCF test time. Results show that the number, size and severity of WECs do not increase linearly (see Fig. 12, 13), a ramped increase seen at the later stages of RCF operation (12–18 h). This could be due to WECs coalescing to form larger crack networks resulting in a ‘weakening’ of the surrounding steel accelerating WEC growth, this being heightened in the event of a sufficient threshold concentration of diffusible hydrogen being reached [44]. Variance in severity is also observed between rollers. This highlights the importance of analysing a representative volume of steel. Differences in the severity between outer and inner roller halves at 18 h are also shown, the outer half having a greater severity than the inner.

Condition monitoring on FAG-FE8 tests [29] has suggested a rapid release of subsurface WECs occurring at ~ 20% outstanding RCF test time before WSF failure. This is proposed to be due to the steel experiencing a failure-free period (no WECs detected), in which energy is absorbed (explained by Barkhausen noise (BN) measurements [54]), a limit being reached with a sudden release of WECs. This failure-free period could be local subsurface transformations that have been observed as ‘crack-free’ dark etching regions suggested to lead to the formation of WEA and subsequently WEC [39]. This investigation shows that WECs do exist during this period before a sudden rupture occurs, ~ 20% outstanding RCF time corresponding to 14.4 h, WECs being recorded between 4 and 12 h.

When comparing the inclusion–WEC interactions recorded at 4–18 h, the inclusions are: (1) consistent in type and size, typically small/short ~ 2–15 μm *D*_Dup_ or *D*-type inclusions, (2) the inclusion–WEC interaction depths are within/close to the zones of maximum subsurface shear stress (*τ*_0, max_ = 92 μm, *τ*_uni, max_ = 145 μm), specifically at the early stages of RCF (4 and 6 h) where initiation is suggested to occur (see Fig. 8), and (3) when visually comparing the inclusion–WEC interactions recorded across 4–18 h, a number of similarities in regard to crack shape/angle and continuity, inclusion type and size, direction of crack propagation and location of inclusion within the WEC network can be observed (see Figs. 9, 10, 16). It is therefore proposed that the WECs recorded in this study were formed as a result of WECs that initiated at NMIs in the subsurface, individual WECs propagating and coalescing at later stages of RCF (12–18 h) to form larger WEC networks. It can also be said that the 19 WECs that did make a connection to the contact surface at 18 h are likely formed as a result of subsurface initiation at inclusions. A number of these surface connections/interactions had very small contact crack volumes and connection to the surface over short axial lengths (see Fig. 6a–c); it is proposed that these connections are not sufficient to drive such extensive WEC networks in the subsurface. Note that a number of these surface connections were in fact very near (< 1 μm) surface ‘interactions’ (see Fig. 6c, f), where under optical microscopy no apparent connection to the contact surface was observed. Further evidence for subsurface initiation comes from the fact that for the 9 fully mapped subsurface WECs at 4 and 6 h, each WEC had at least one or multiple inclusion–WEC interactions. Additional evidence to support subsurface initiation of WECs by NMIs is shown through the visual comparison of typical recorded WECs across the 4–18-h tests (see Fig. 16). As it can be seen the initial shape and propagation route of WECs at 4 and 6 h follow a close link to those WECs recorded at 12 and 18 h, this is in conjunction with the fact that the inclusion–WEC interactions are also closely linked by the similarities discussed above. At 4 and 6 h, WECs are found to initially resemble butterfly cracks, which propagate into ‘star-like’ cracks with forking of the butterfly crack tips. This ‘star-like’ crack shape can be seen to fit a number of the WECs found at 12 and 18 h as shown in Fig. 16. It is thus proposed that the WECs recorded at 4 and 6 h are the early initiation stages of WECs that subsequently propagate and evolve into the large WEC networks recorded at 18 h. The proposed evolutionary stages of WEC initiation are shown in Fig. 16g.

The outer and inner roller halves have shown to significantly influence the propensity and size of WEC formations, the outer half being dominant over the inner (see Table 3, Figs. 11, 12, 13 and Video 2). Slip has been shown to influence the formation of WECs in both FAG-FE8 and three ring roller micropitting rig (MPR) tests using the same ‘special’ oil known used in this study [29, 55, 56], where evidence for the influence of negative slip being more dominant in WSF over positive slip is provided [56]. More recently the influence of slip on WEC formations has also been shown in a two-disc test rig set-up, where again negative slip showed dominance in WEC production in contrast to positive [57]. This dominance has been attributed to higher material stressing, lowered fracture mechanic properties under alternating load and preferential surface crack propagation due to the traction force and surface motion vectors pointing in the same direction in negative slip as opposed to positive [57]. It is proposed that negative slip results in the compressive closure of cracks enhancing the crack rubbing mechanism for WEA formation [56]. The localisation of the WECs recorded across 4–18 h is more densely populated in the 2–3 mm (outer) and 8–9 mm (inner) zones across the roller (see Fig. 12, Video 2). These zones correspond to areas of high slip energy (PVmax, the product of contact pressure P and slip velocity V, MPa ms^−1^, see Fig. 12), slip energy taking into account asperity contact (P_c_V value which takes into account the asperity contact pressure P_c_) and asperity friction accumulation *e*_*a,c* max_ which relates the regeneration time span between consecutive contact load cycles on tested WEC lives and the specific frictional energy input into the a surface during the contact load cycle [58]. Slip energy criteria have been linked to WSF, WSF occurring at areas of greatest PVmax. These areas have also been found to coincide with zones of highest concentrations of hydrogen [50, 59, 60]. Supporting evidence for the slip energy criterion has been shown on FAG-FE8 tests where WECs appeared firstly at areas of high frictional energy, this also being demonstrated in tests using angular contact ball bearings [29, 58]. A number of inclusion–WEC interactions were also recorded during fine serial sectioning corresponding to the 2–3-mm zone of high slip energy dissipation and asperity accumulation (see Fig. 10). Further progression of the slip energy criteria concept has been developed based upon information from different test rigs, using normal contact load and representing the slip energy criteria per film thickness sheared (N V/λ, N ms^−1^) to determine a threshold for WEC formation in most roller bearing configurations [21, 61]. It is postulated that this threshold could exist due to the fact that sliding energy generates local flash temperatures influencing the tribochemical reactions taking place at nascent surfaces [62]. Limitations, however, do exist as this criterion does not take into account the lubricant formulation. Evidence for the degree of boundary lubrication (the range of *λ*) controlling the propensity for WEC formation is also suggested, more WECs forming for more severe boundary regimes (*λ* in the range of 0.06–0.7) [56]. No WECs were found in the raceway washers, with no evidence of WEC formations being observed in the zones corresponding to high slip energy dissipation or asperity friction accumulation. It is noted that the asperity friction energy accumulation is greater in the washers than the rollers, where energy dissipation is greater in the inner raceway than the outer (see Fig. 6, [58]). This is contradictory to the result seen in this study, this discrepancy not being understood.

#### WEA Volume

Metallographic analysis has shown that the volume of WEA associated with cracks increases for longer RCF test operation. Through quantitative WEA analysis, the average WEA volume (μm^3^) and area (μm^2^) associated with cracks increased between 4 and 18 h, a ramped increase found between 12 and 18 h (see Fig. 14a). Analysis also reveals that for greater axial WEC lengths a significant increase in the associated WEA volume is found (Fig. 14b). It is proposed that the evidence found in this study supports the theory of crack rubbing/beating in the formation of WEAs [36]. As WECs grow and propagate during RCF operation, further crack rubbing/beating occurs at the newly formed crack faces, larger cracks having a greater amount of ‘free’ crack faces available for extended crack rubbing/beating to occur. This can also be exhibited in Fig. 16 where it can be seen that the amount of WEA associated with the cracks increases across 4–18 h, an increase being observed between 12 and 18 h. Further to this, by visually observing the mapped WECs at 4 and 6 h (see Fig. 8) a decrease in WEA volume is seen at the start and ends of the WEC, i.e. the extreme tips when visualised as a 3D network. Taking for example the 6-h WEC in Fig. 8, where it is proposed that the inclusion is the site of initiation; it can be seen that the volume of WEA is greater around the inclusion site (see Fig. 8g) than at the end ‘tips’ where branching/forking has occurred (final stages of propagation) where it is proposed that less time has been available for crack rubbing/beating. This is also exhibited in two videos through serial sectioning of an 18.5-h FAG-FE8 test previously conducted by the authors [24]. This point is strengthened through WEA analysis where it has shown that the average amount of WEA (μm^2^) is less at the tips (1st (start) and 5th (end) measurements) than at the centre (3rd (middle) measurement) (see Fig. 14a). As discussed, a large number of near-surface WEA/WECs were recorded at 18 h (see Table 3, Fig. 7). A number of these features were found not associated with a crack, leading to the conclusion that near-surface WEAs do not seem to require a crack to form WEA. However, note that in this study only optical microscopy has been used in the classification of WEAs associated with cracks, further analysis using SEM to confirm the non-existence of small cracks that may be present inside the WEA regions should be conducted.

**Fig. 18:**
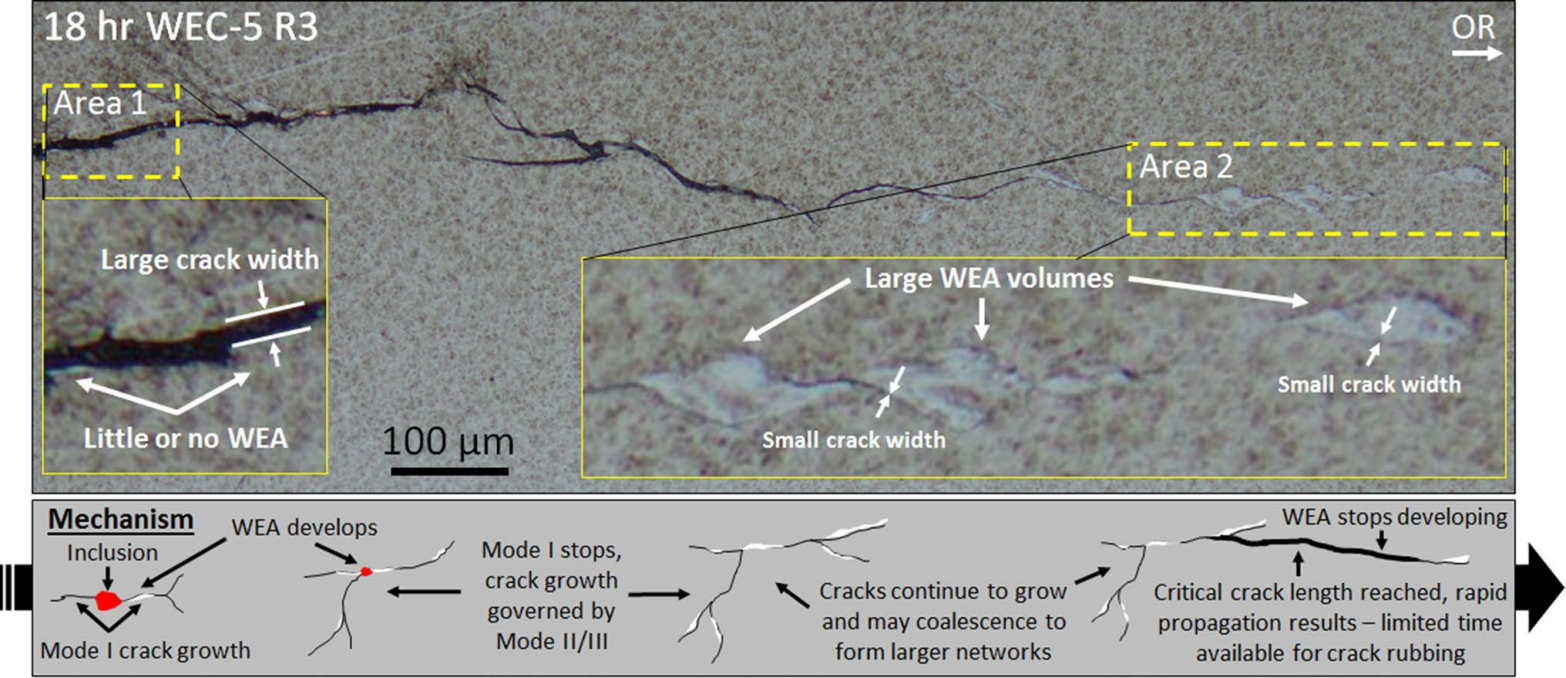
Optical image of a WEC demonstrating the influence of crack width on the generation of WEAs. Area 1 shows a large crack width, and Area 2 shows a small crack width, with respective magnified optical images. The proposed mechanism for the development of WEA w.r.t crack width is shown. Over-rolling (OR) direction left to right

Through metallographic analysis, it is indicated that the angle of crack propagation, crack width and zone of maximum subsurface shear stresses can influence the degree of WEA generated. WEA analysis has shown that the amount of WEA associated with a crack increases the nearer to 0° or perpendicular to the axis of compression a crack propagates (see Fig. 14c). A vertical crack ± 90° parallel to the axis of compression is found to have very little WEA associated (see Fig. 14c). This is also exhibited clearly in the 18-h crack shown in Fig. 17. As suggested by others [36], it is proposed that a vertically branching crack will be subjected to a much lesser amount of crack rubbing. The localisation of strain has been found to be strongly reliant on crack orientation in relation to stress [63]. High strain rate compressive tests have shown regions of WEA [64], this being in comparison with equivalent tensile tests; as a result it is proposed that crack rubbing/beating under RCF shear stresses or compressive loading results in WEA formations and thus adiabatic shearing is an unlikely cause [65]. The crack width also appears to influence WEA formations. For example, in Fig. 18 it is seen that very little or no WEAs are associated with sections (Area 1) of the WEC that have large crack width when compared to crack faces that are close together (Area 2). It is proposed that for adjacent crack faces that are further apart, less action is available for crack rubbing/beating. This is not to say, however, that these particular areas of the WEC network would have not been associated with WEAs at some point during operation. The proposed mechanism (see Fig. 18) to explain this is as follows: (1) inclusion initiation of butterfly and ‘star-like’ WECs with subsequent generation of WEAs due to crack rubbing/beating. (2) WECs propagate and WEAs continue to develop. (3) Short crack growth from inclusions/butterflies by Mode I loading [66] stops and further growth is governed by Mode II/III shear loading if the Mode II/III stress intensity factor threshold is surpassed [66], WECs may propagate and coalesce to form larger networks. Until a critical length is reached, crack growth rate may be slow, where once exceeded rapid propagation results under applied stress. (4) Due to the rapid growth of the crack and crack volume, the time and action available for WEA development is alleviated and thus a reduction or non-existent presence of WEA is seen. In the event of hydrogen diffusion, hydrogen acts to decrease the Mode I/II stress limits for crack growth and propagation [66, 67], it may be reasoned that this step increase in WEC formations is due to a threshold concentration of hydrogen being reached for a decrease in Mode II crack growth [44].

Analysis also indicates that the zone of maximum subsurface shear stresses influences WEA generation. This is most clearly exhibited in WECs recorded at 18 h. For example, WEC-2 R1 in Fig. 17 shows that a greater amount of WEA exists within and in the regions around the zone of maximum subsurface shear stresses (*τ*_0, max_ = 92 μm, *τ*_uni, max_ = 145 μm).

#### Inclusion–WEC Interactions and Steel Cleanliness

Eighty-two NMIs were recorded during the metallographic analysis; 62 were ranked with a high likelihood of WEC initiation (rank 1 or 2). Most inclusions were found to be small sized (~ 2–15 μm (diameter) and ~ 4–21 μm in axial length) globular duplex inclusions (globular manganese and/or calcium sulphide surrounding aluminate) (*D*_Dup_) and globular oxide inclusions (*D*). At the early infant stages of WEC formation (4 and 6 h), D_Dup_ and D-type inclusions were found to interact with the WECs, EDX analysis of inclusions at these stages being either Al_2_O_3_ or MnS surrounding Al_2_O_3._ Inclusion types found to interact with the large WEC networks found at the later stages of RCF duration (18 h) are consistent with those found at the early infant stages. This agrees with the findings found from the 18.5-h RCF test in [24] that found 49 NMIs and 41 rank 1 or 2 NMIs in 5 fully mapped WECs, these predominantly being small sized (~ 2–15 μm) *D*_Dup,_ *D*_DupTi(C,N)_ and *D*-type inclusions. The oxide encapsulations are responsible for hardness discrepancy with the martensite matrix, induced tensile residual stresses due to differing coefficients of thermal expansion and weak coherence/de-bonding of the oxide and matrix [9, 68, 69]. The majority of the inclusion–WEC interactions were recorded at a depth of ~ 50–200 μm, this being consistent with the depth of high subsurface shear stresses (*τ*_0, max_ = 92 μm, τ_uni, max_ = 145 μm) (see Fig. 10).

Steel cleanliness analysis indicates that the raceway is ‘cleaner’ than the rollers (*C*_*i*_ = 1.5 (standard) and *C*_*i*_ = 344.1 (non-standard) for the rollers and (*C*_*i*_ = 0.2 (standard) and *C*_*i*_ = 35.6 (non-standard) for the raceway). The ‘cleaner’ raceway would therefore have fewer inclusions available to initiate cracks, which could explain why no WECs were recorded. The lower cleanliness and therefore greater density of inclusions in the rollers would also lead to an increased propensity for WECs to initiate and coalesce to form more extensive networks, this being elevated if inclusions lie in critical locations. It is important to note the significant increase in *C*_*i*_ when only counting inclusions recorded under the thresholds set by the ISO 4967-B standard [48] and when counting inclusions recorded outside of these domains; however, the ratio difference in cleanliness between the roller and raceway remains similar. Small/short inclusions have been found to be dominant in initiating and interacting with WECs. Thresholds set by the standard do not factor in these small/short inclusions, comparisons of the *C*_*i*_ highlighting the potential limitations of the standard when considering steels used in WTGBs. These limitations are currently being investigated and will be presented in a future study by the authors.

## Conclusions

1. Metallographic analysis has been used to map white etching crack (WEC) damage in RCF-tested bearings in standard 100Cr6 steel. For the first time, this study has captured the evolution of WEC formation, using serial sectioning methods to investigate the formation mechanisms of WECs in FAG-FE8-tested bearings under non-hydrogen charged conditions.
2. From the characteristics, location and apparent evolution of WECs over the increasing test durations, macro- and serial sectioning has enabled further verification of the author’s original revelations that (1) WECs can initiate and propagate entirely within the subsurface and (2) the frequent interaction with small/short inclusions strongly indicates that WECs can often be initiated by non-metallic inclusions. The inclusion interactions are ~ 2–15 μm in the circumferential direction, and ~ 4–21 μm in axial length, being globular sulphides (*D*_sulf_), globular duplex inclusions (globular manganese and/or calcium sulphide surrounding aluminate) (*D*_Dup_) and globular oxide inclusions (*D*). *D*_Dup_ and *D*-type inclusions are found to interact with the WECs at the early infant stages of WEC formation, inclusions being either Al_2_O_3_ or MnS surrounding Al_2_O_3._ Inclusion types found to interact with large WEC networks found at the later stages of RCF duration are consistent with those found at the early infant stages. The cleanliness of the roller and raceway were found to be significantly different, the rollers having a much lower cleanliness than the raceway, which may help explain why no WECs were found in the raceway sections. 
3. Detailed analysis of WEC characteristics across the test durations, such as quantification of the amount of WEA microstructural change associated with the WECs over the test durations, and also within certain planes of the WEC, has provided supporting evidence for the crack being a prerequisite to WEA, where a possible mechanism for this is crack face rubbing. Conversely to this mechanism, in the samples exposed to most test duration, numerous small very near-surface WEAs were also found without any visible crack; thus, evidently the formation of the microstructural change to WEA does not require the presence of a crack. Further analysis including SEM should be carried out, however, to also confirm the non-existence of small cracks associated with WEA as only light optical microscopy has been used in this study.
4. An interesting finding is a heterogeneous distribution of WEC formation occurred in the bearing rollers, most WECs forming in a relatively limited zone corresponding to where the largest energy dissipation occurs, agreeing with recent literature observations.

### Electronic supplementary material

Below is the link to the electronic supplementary material.

Supplementary material 1 (AVI 14475 kb)

Supplementary material 2 (AVI 495188 kb)

## Figures and Tables

**Fig. 19:**
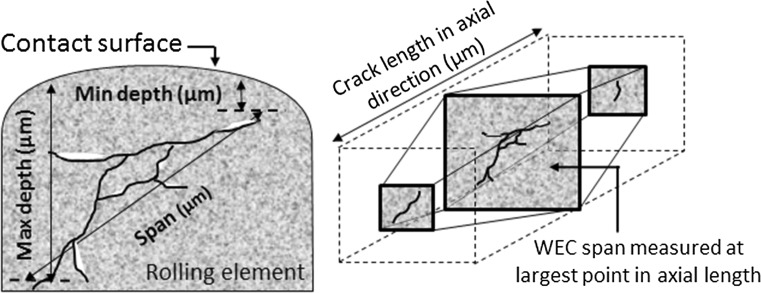
Schematic illustrating the measurements of WEC dimensions

## Appendix A

The minimum oil film thickness (hmin) between the rolling elements and raceway washer was calculated using Hamrock–Dowson viscous-elastic calculation [46, 47], see Eq. (1).

1 $$ h_{ \hbox{min} } = \, 3.63{\text{U0}}^{.68} {\text{G}}^{0.49} {\text{W}}^{ - 0.073} \left( {1 - e^{{ - 0.68\,{\text{k}}}} } \right) $$

where *h*_min_ minimum film thickness [m], *U* dimensionless speed parameter, *uη*_0_/(*E’R*), *G*: dimensionless material parameter, *αE’, W*: dimensionless load parameter, *N*/(*E’R* ^2^), *u*: mean lubricant entrainment speed, [m/s], *η0*: viscosity at atmospheric pressure of lubricant, [Pa s], *E*’: effective elastic modulus, [30], *R*: reduced radius of curvature, [m], *α*: pressure–viscosity coefficient, [Pa^−1^], *N*: normal load, [N], k: ellipticity parameter defined as: *k* = a/b, where ‘*a*’ is the semiaxis of the contact ellipse in the transverse direction [m] and ‘*b*’ is the semiaxis in the direction of motion [m]. For line contact, *k* = ∞.

The initial lambda ratio (λ) was then calculated using Eq. (2), where Rq, 1 and Rq, 2 are the rms roughness values of the two contact surfaces.

2 $$ \lambda = \, h_{ \hbox{min} } /\left( {{\text{Rq}},\,1^{2} + {\text{ Rq}},\,2^{2} } \right)^{1/2} . $$

## Appendix B

### Rank 1

Strong evidence for butterfly initiated WECs.

1. Orientation of wings (+ ve or − ve) being consistent with the wings of independent butterflies found in the serial sectioning analysis (not in the WEC network). + ve or − ve wing orientation is determined by the over-rolling direction, a second pair of wings being formed upon reversal of the over-rolling direction after a certain period [70].
2. The crack/wing angle, this being mainly ~ 45º with a range of 0–60° for typical butterfly formations [4, 5].
3. If the microstructural change morphology associated with the crack/wing is of a typical ‘classic wing-like pattern’.

### Rank 2

Possible butterfly initiation or strong likelihood of WEC initiation independent of butterfly.

1. This is the case when crack initiation features are observed; however, the characteristics of butterfly formation as stated in Rank 1 type damage features are not observed.

### Rank 3

Crack has passed through the inclusion during crack propagation; inclusion can therefore potentially aid in the propagation; however, the inclusion is not involved in the initiation process of a crack.

1. Orientation of the crack when passing through the inclusion was in a radial or near radial direction.
2. The inclusion interaction location is somewhere along one of the radial (depth direction) cracks of the WEC. Also microstructural change may not be observed around the crack.
3. The inclusion interaction depth is in an area of low subsurface shear stress.
4. The inclusion is small and is located inside a zone of considerable microstructural change with no crack visibly connecting to the inclusion.

## Appendix C

See Fig. 19.
